# Supplementary material for: Pyroptosis leads to loss of centrosomal integrity in macrophages
Source: Cell Death Discov. 2024 Aug 8;10:354. doi: 10.1038/s41420-024-02093-1 (PMC11310477; doi:10.1038/s41420-024-02093-1)
Supplement: Supplementary file 1 — Supplementary figures [file 41420_2024_2093_MOESM1_ESM.pdf]

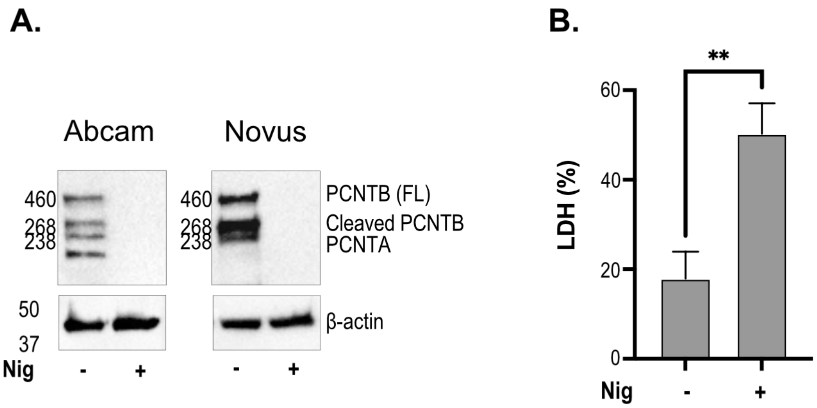

Fig. S1 Nigericin treatment of THP-1 cells results in loss of centrosomal integrity.
THP1^{ATCC} cells were left untreated or treated with nigericin (10 µM) for 45 min to activate the NLRP3 inflammasome (A-B, N=3). Lysates were analyzed by WB for PCNT expression using two different antibodies, Abcam (ab4448) or Novus PCNT antibody (NB100-61071) as well as loading control β-actin (A). Cell death was measured by LDH assay and shown as percentage relative to total cell death (B). Representative blot of n=3

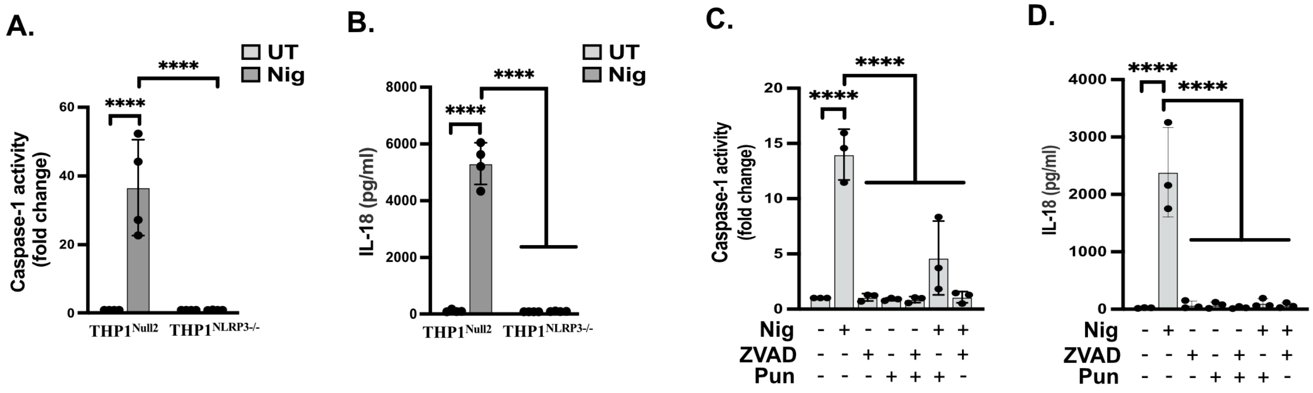

Fig.S2 Caspase-1 activity and IL-18 release in THP1 with impaired NLRP3 activation (Related to figure 2).

THP1^{Null2} and THP1$^{NLRP3-/-}$ cells were stimulated with nigericin (10 μM, 45 min) (A-B, N=4). THP1^{Null2} cells were left untreated or treated with punicalagin (50 μM, 15 min) or ZVAD (50 μM, 40 min), after which cells were stimulated with nigericin (10 μM, 45 min) to activate the NLRP3 inflammasome (C-D, N=3). Caspase-1 activity was measured by caspase-1 assay and shown as fold change relative to control (A, C). IL-18 was measure by ELISA (B, D).

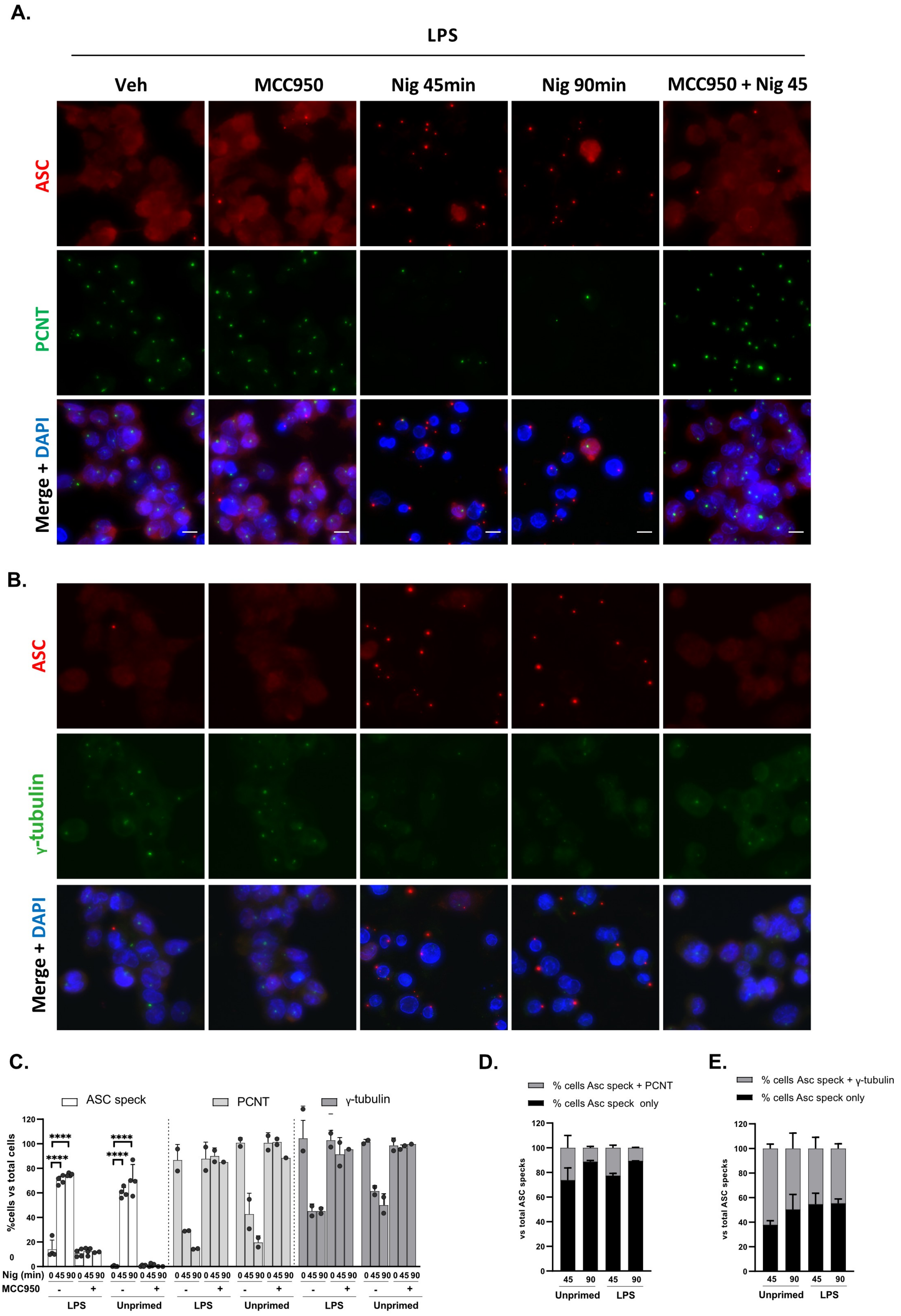
A.
LPS
Veh
MCC950
Nig 45min
Nig 90min
MCC950 + Nig 45
ASC
PCNT
Merge + DAPI
B.
ASC
γ-tubulin
Merge + DAPI
C.
ASC speck
PCNT
γ-tubulin
%cells vs total cells
****
Nig (min)
MCC950
LPS
Unprimed
D.
% cells Asc speck + PCNT
% cells Asc speck only
vs total ASC specks
Unprimed
LPS
E.
% cells Asc speck + γ-tubulin
% cells Asc speck only
vs total ASC specks
Unprimed
LPS

Fig.S3 Priming does not alter PCNT loss caused by nigericin in THP1 cells.

THP1 ^{ATCC} cells were primed with LPS (1 µg/ml, 4 h) or not, then treated with MCC950 (10 µM, 15 min), after which cells were stimulated with nigericin (10 µM) for 45 or 90 min (A-E, N=3). Immunofluorescence was used to analyze PCNT, γ-tubulin and ASC (A, B). Percentages of ASC speck, PCNT or γ-tubulin positive cells relative to total cells were calculated (C). Percentages of both ASC speck and PCNT or only ASC speck positive cells in total ASC positive cells (D) and of both ASC speck and γ-tubulin or only ASC speck positive cells in total ASC positive cells (E) were calculated. 300 cells were analyzed per experiment. Independent experiments, N=3.

Fig S4

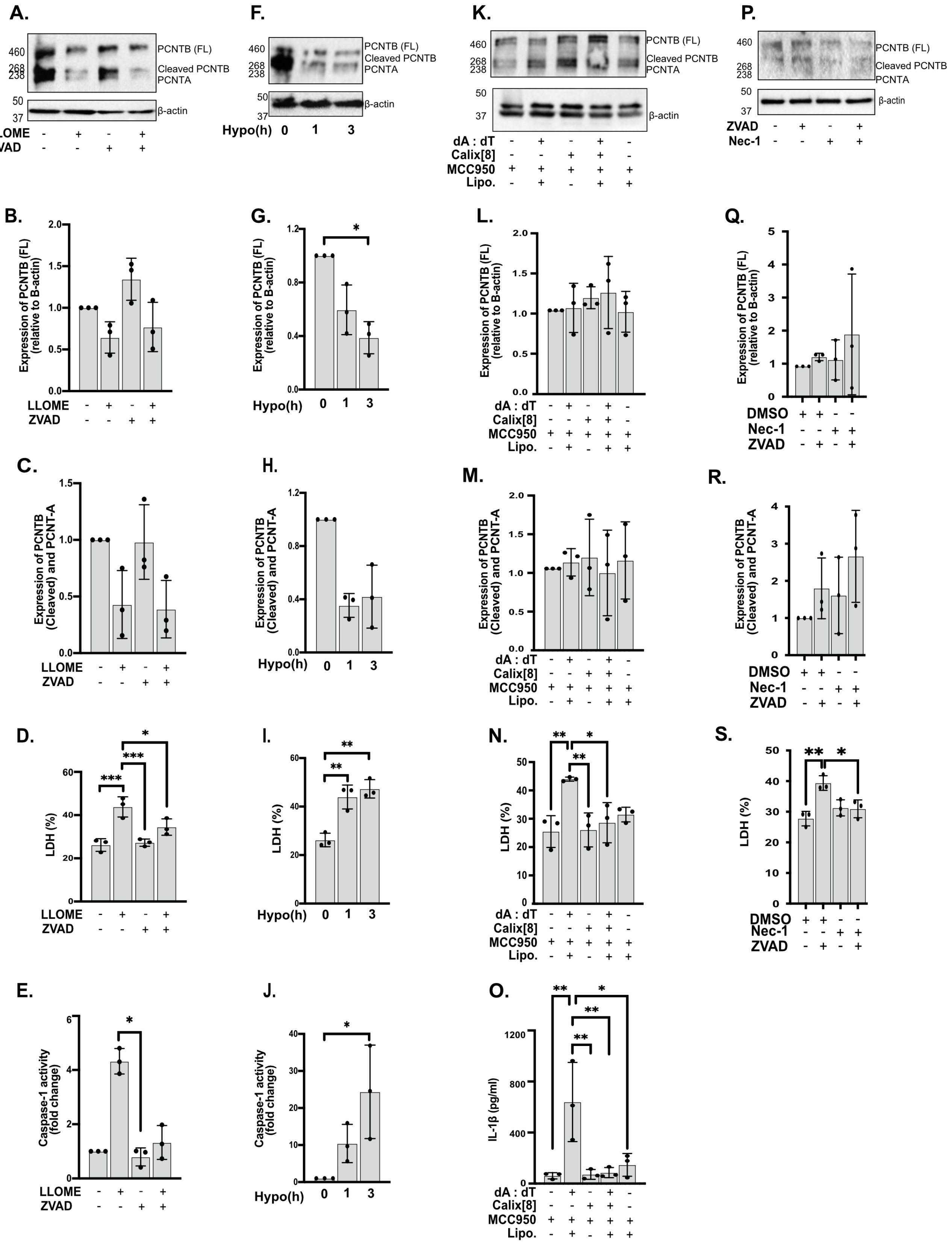
A.
PCNTB (FL)
Cleaved PCNTB
PCNTA
β-actin
LLOME
ZVAD
F.
Hypo(h) 0 1 3
K.
dA : dT
Calix[8]
MCC950
Lipo.
P.
ZVAD
Nec-1
B.
Expression of PCNTB (FL) (relative to B-actin)
G.
L.
Q.
DMSO
C.
Expression of PCNTB (Cleaved) and PCNT-A
H.
M.
R.
D.
LDH (%)
I.
N.
S.
E.
Caspase-1 activity (fold change)
J.
O.
IL-1β (pg/ml)

Fig.S4 PCNT protein levels are shown in response to NLRP3, AIM 2 and necroptosis activation. THP1^{ATCC} cells were left untreated or treated with ZVAD (50 μM, 40 min), then treated with LLOMe (1 mM, 1 h) to activate the NLRP3 inflammasome (A-E, N=3). Lysates were analyzed for PCNT as well as loading control β-actin (A). Relative expression of full length PCNT-B and cleaved PCNT-B/PCNT-A compared to the β-actin was quantified respectively (B, C). Cell death was measured by LDH assay and shown as percentage relative to total cell death (D). Caspase-1 activity was measured by caspase-1 assay and shown as fold change relative to control (E). THP1^{ATCC} cells were left untreated or treated with a hypotonic buffer (Hypo) for 1 or 3 h to activate the NLRP3 inflammasome (F-J, N=3). Lysates were analyzed for PCNT as well as loading control β-actin (F). Relative expression of PCNT (G, H), cell death (I) and caspase-1 activity (J) were quantified or measured as described above. THP1^{ATCC} cells were primed with LPS for 4 h, then left untreated or pre-treated with 4-Sulfocalix[8]arene (Calix[8]) 10 μM for 15 min and followed by transfection with poly dA:dT 1 μg. This was done in the presence of MCC950 10 uM (K-O, N=3). Relative expression of full length PCNT-B and cleaved PCNT-B/PCNT-A compared to the β-actin was quantified respectively (L, M). Cell death (N) and IL-1β (O) were measured. THP1^{ATCC} cells were primed with LPS for 4 h, then left untreated or pre-treated with Nec-1 50 μM for 15 min and followed by ZVAD (50 μM) treatment for 24 hr to induce the necroptosis (P-S, N=3). Relative expression of full length PCNT-B and cleaved PCNT-B/PCNT-A compared to the β-actin was quantified respectively (Q, R). LDH release was measured (S).

Fig S5

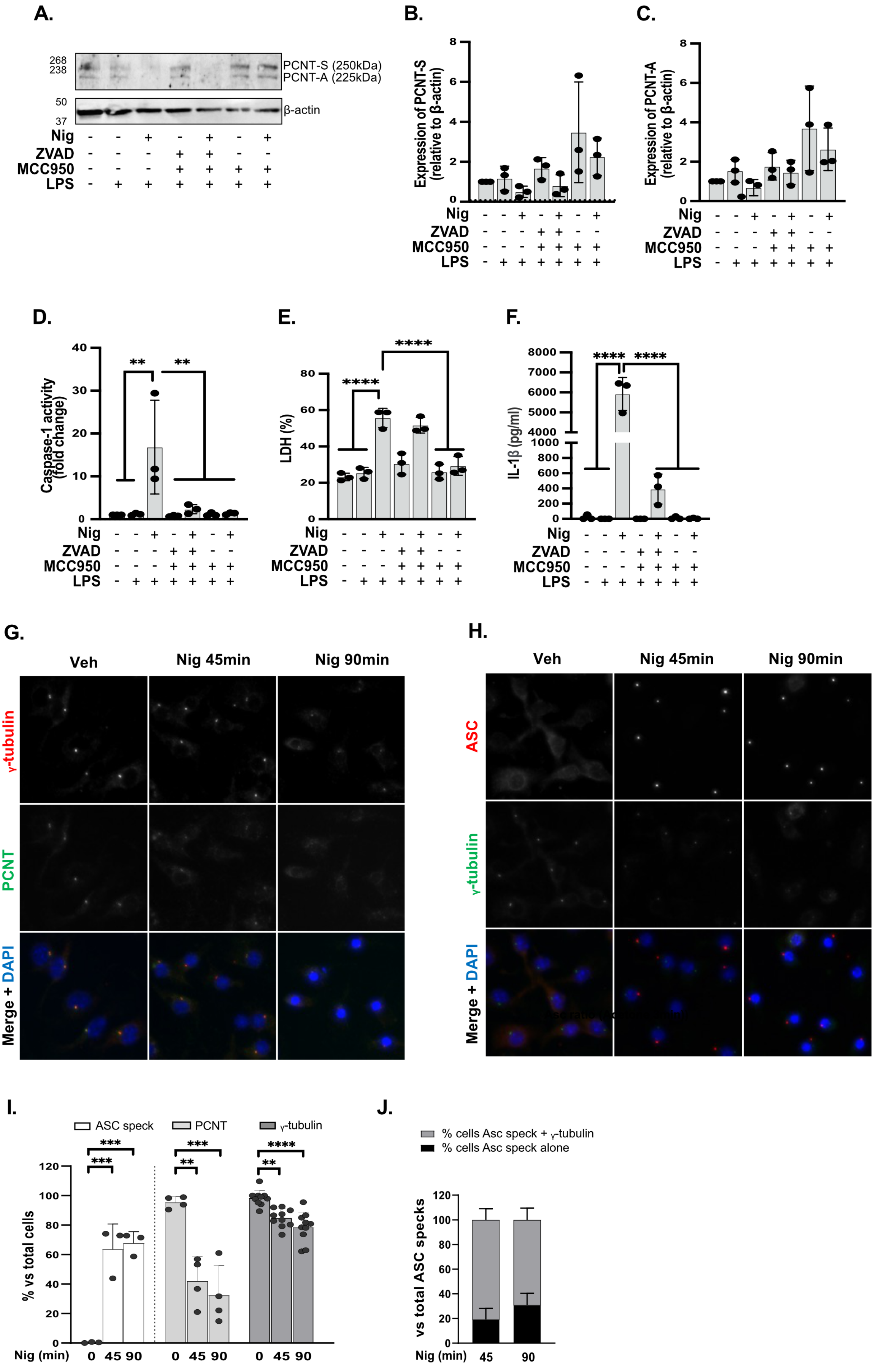
A.
268
238
PCNT-S (250kDa)
PCNT-A (225kDa)
50
37
β-actin
Nig
ZVAD
MCC950
LPS
B.
Expression of PCNT-S (relative to β-actin)
C.
Expression of PCNT-A (relative to β-actin)
D.
Caspase-1 activity (fold change)
E.
LDH (%)
F.
IL-1β (pg/ml)
G.
Veh
Nig 45min
Nig 90min
γ-tubulin
PCNT
Merge + DAPI
H.
ASC
I.
ASC speck
Nig (min)
% vs total cells
J.
% cells Asc speck + γ-tubulin
% cells Asc speck alone
vs total ASC specks

Fig.S5 Nigericin triggers PCNT loss in BMDMs.

LPS (1 μg/ml, 4 h) primed BMDMs were left untreated or treated with MCC950 (10 μM, 15 min) or ZVAD (50 μM, 40 min), then cells were stimulated with nigericin (10 μM, 45 min) (A-F, N=3). Lysates were analyzed for PCNT as well as loading control β-actin by western blot (A). Relative expression of full length PCNT-S (250 kDa) and PCNT-A (225 kDa) compared to the β-actin was quantified respectively (B, C). Cell death (D), caspase-1 activity (E) and IL-18 (F) were measured. LPS (1 μg/ml, 4 h) primed BMDMs were left untreated or treated with nigericin (10 μM) for 45 or 90 min to activate the NLRP3 inflammasome (G-J). Immunofluorescence was used to analyze PCNT, γ-tubulin and ASC (G, H). Percentages of ASC speck, PCNT or γ-tubulin positive cells relative to total cells (I) and both ASC speck and γ-tubulin or only ASC speck positive cells in total ASC positive cells (J) were calculated. 300 cells were counted and analyzed per experiment. Independent experiments, N=3.

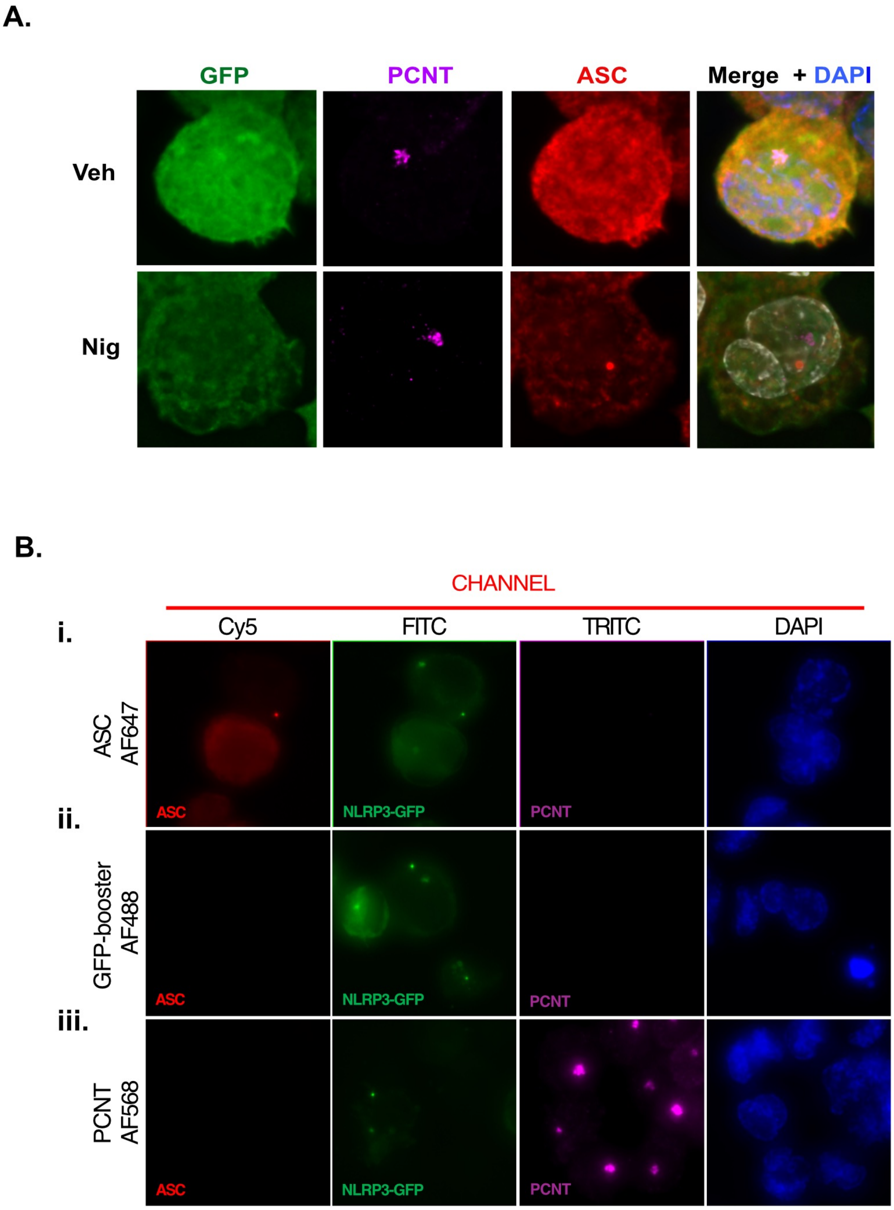

Fig.S6 GFP alone does not re-locate to the centrosome with nigericin stimulation in THP1 cells. GFP-THP1 cells were left untreated or treated with nigericin (10 µM, 45 min) (A-B, N=3). Immunofluorescence was used to analyze GFP, PCNT and ASC (A, B). Immunofluorescence controls are shown in Fig S6 (B) for different channels settings and for experiments in Fig 3. Absence of PCNT staining in (i) shows absence of signal transfer between Cy5 and FITC to the TRICT channels; absence of ASC and PCNT stain in (ii) shows absence of signal transfer from FITC to the Cy5 and TRICT channels; absence of ASC stain in (iii) shows absence of signal transfer between TRICT and FITC to Cy5 as well as no signal transfer from TRICT to FITC channels.

Fig S7

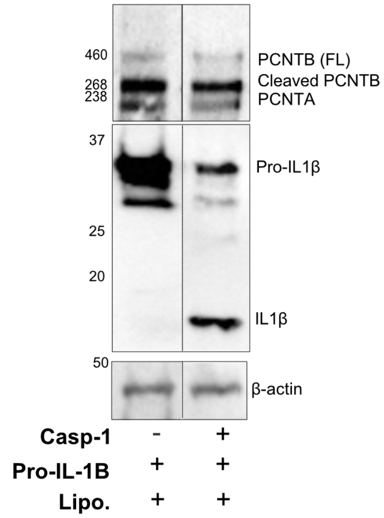

Fig.S7 PCNT loss is not directly mediated by caspase-1.
HEK293 cells were co-transfected with pro-IL-1B and caspase-1, and PCNT protein levels were assessed by WB (N=3). Expression of pro-IL-1β (32kDa) and its cleavage to mature form (IL-1β; 17kDa) was measured by western blot. β-actin was used as a loading control.

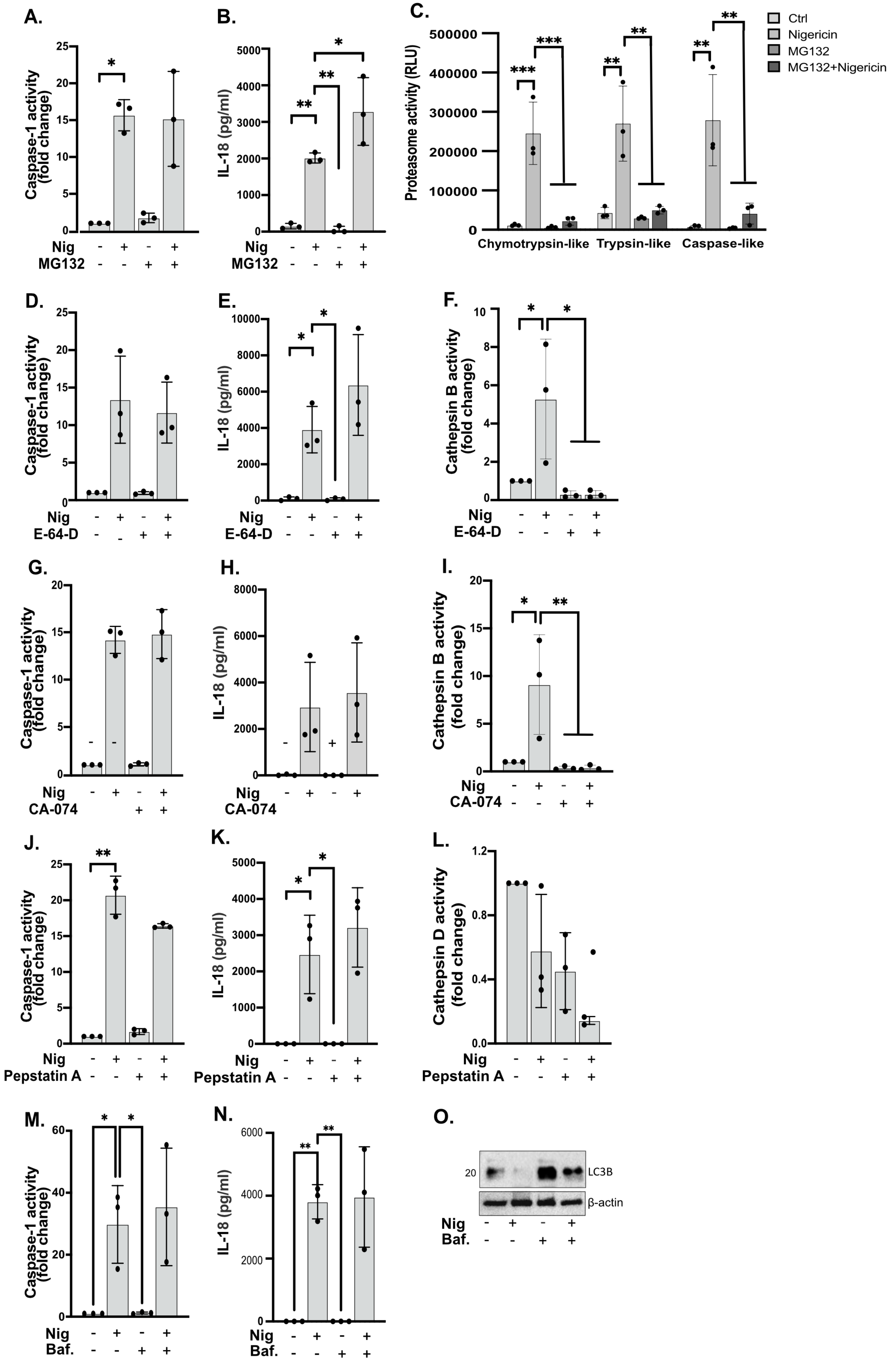

A.
Caspase-1 activity (fold change)
Nig
MG132
B.
IL-18 (pg/ml)
Nig
MG132
C.
Proteasome activity (RLU)
Chymotrypsin-like
Trypsin-like
Caspase-like
Ctrl
Nigericin
MG132
MG132+Nigericin
D.
Caspase-1 activity (fold change)
Nig
E-64-D
E.
IL-18 (pg/ml)
Nig
E-64-D
F.
Cathepsin B activity (fold change)
Nig
E-64-D
G.
Caspase-1 activity (fold change)
Nig
CA-074
H.
IL-18 (pg/ml)
Nig
CA-074
I.
Cathepsin B activity (fold change)
Nig
CA-074
J.
Caspase-1 activity (fold change)
Nig
Pepstatin A
K.
IL-18 (pg/ml)
Nig
Pepstatin A
L.
Cathepsin D activity (fold change)
Nig
Pepstatin A
M.
Caspase-1 activity (fold change)
Nig
Baf.
N.
IL-18 (pg/ml)
Nig
Baf.
O.
20
LC3B
β-actin
Nig
Baf.

Fig.S8 Proteasome, CathepsinB and Cathepsin D activities during the NLRP3 activation in THP1 cells (Related to figure 7).

PMA differentiated $THP1^{ATCC}$ cells were left untreated or treated with MG132 (10 µM, 2 h), or E-64-D (20 µM, 2 h), or Ca-074Me (50 µM, 15 min), pepstatin A (10 µM, 15 min) or bafilomycin A1 (100 µM, 15 min) before stimulation with nigericin (10 µM, 45 min) to activate the inflammasome. Activity of released caspase-1 and IL-18 release in the presence of nigericin and the different inhibitors was measured by luminescence caspase-1 assay (A, D, G, J, M) or ELISA (B, E, H, K, N). Proteasome activity was measured, and luminescence was recorded as relative light units (RLU) (C). The activities of cathepsin B and cathepsin D were measured and shown as fold change from the untreated cells control for all experimental groups (F, I, L). LC3B expression was assessed by WB to determine efficiency of bafilomycin A1 treatment (O).
