# Supplementary material for: Pyroptosis leads to loss of centrosomal integrity in macrophages
Source: Cell Death Discov. 2024 Aug 8;10:354. doi: 10.1038/s41420-024-02093-1 (PMC11310477; doi:10.1038/s41420-024-02093-1)

Fig1

Time course

PCNT lysates

N=1

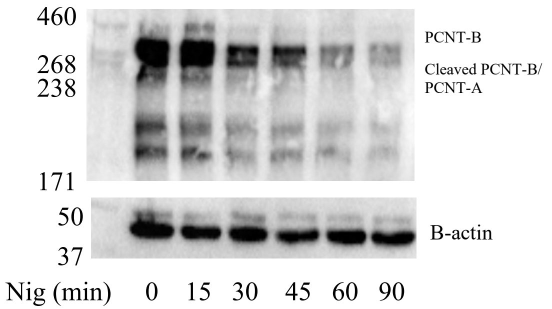

N=2

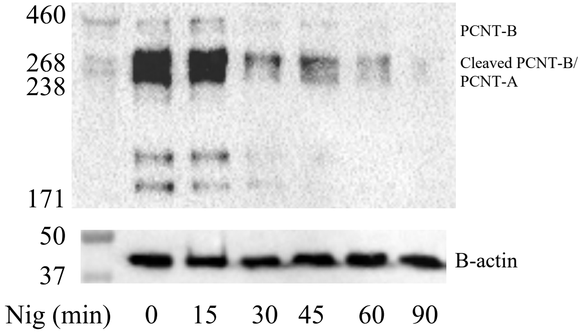

N=3

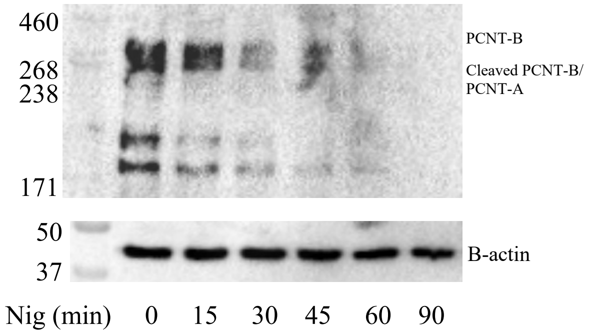

PCNT supernatants

N=1

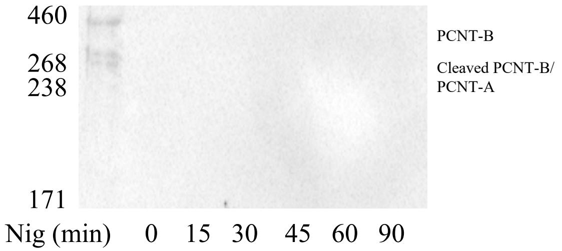

N=2

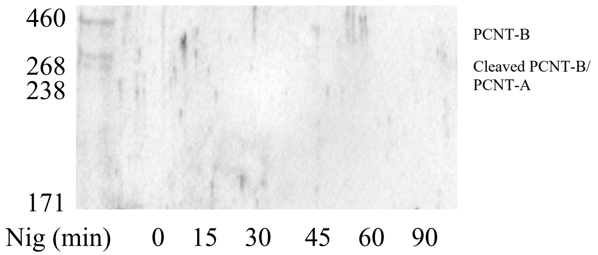

N=3

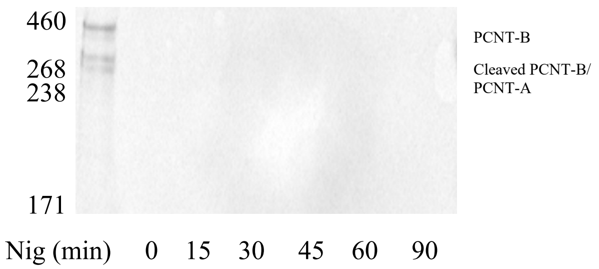

Fig1

Time course

PCNT whole well lysates

N=1 ATCC

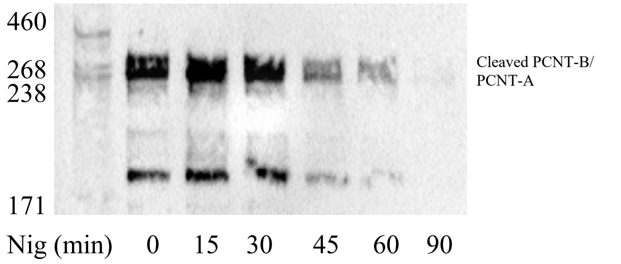

N=2 ATCC

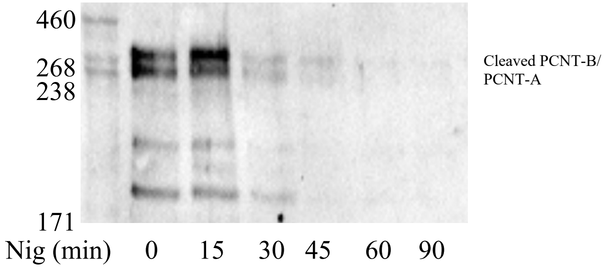

N=3 Null2

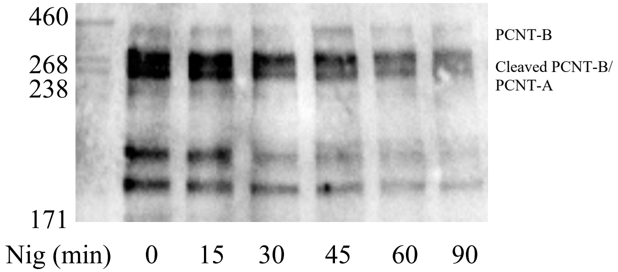

Fig1

Time course

gammatubulin whole well lysates

N=1

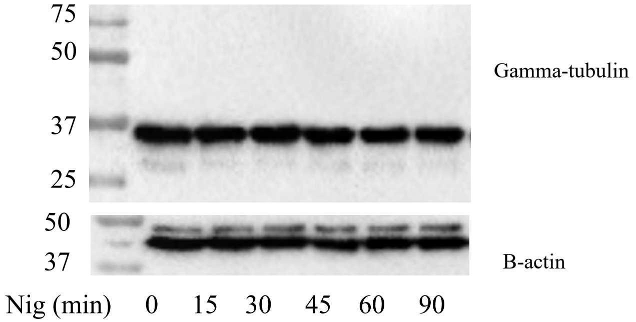

N=2

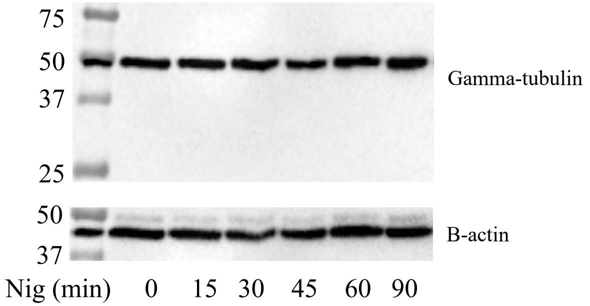

N=3

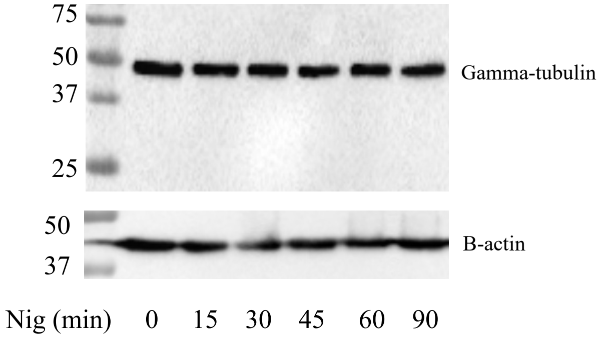

Fig1

Time course

gammatubulin lysates

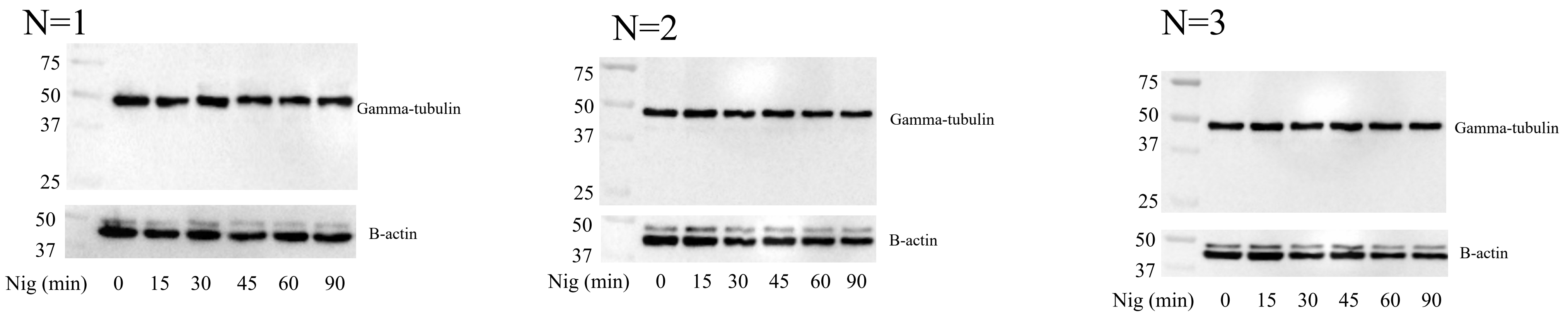

gammatubulin supernatants

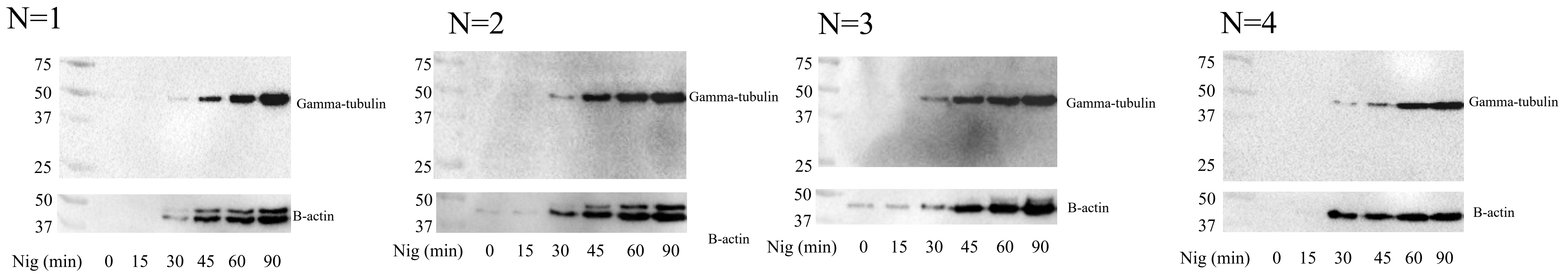

Fig1

lysates and supernatants without centrifugation

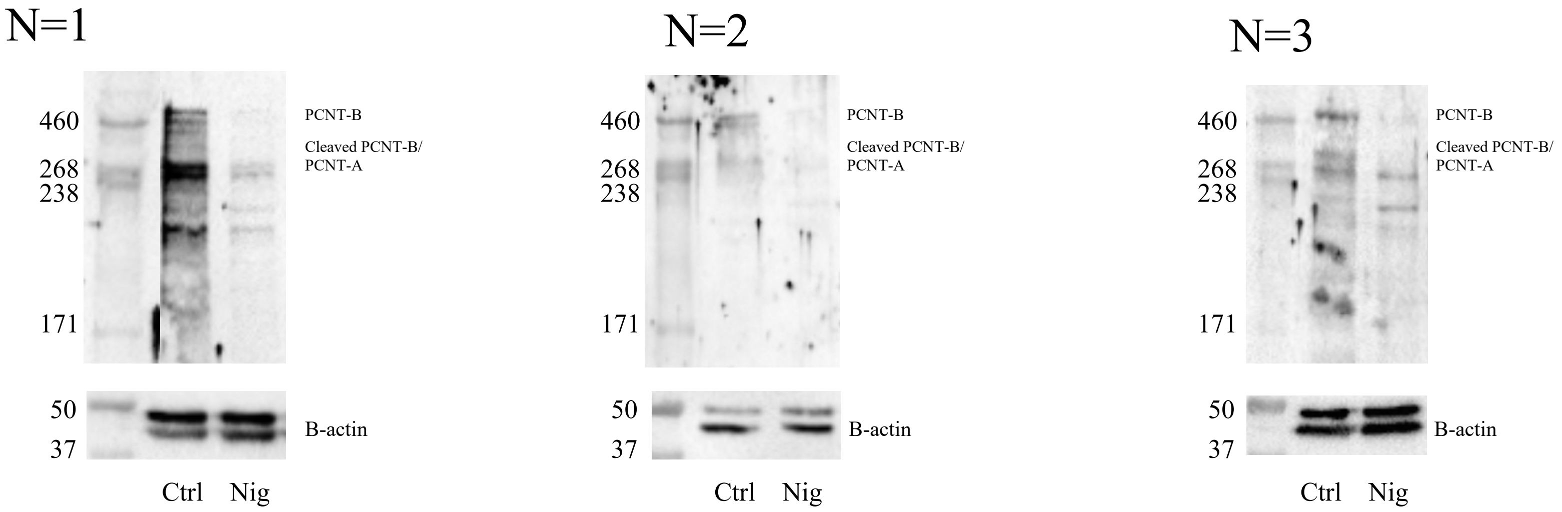

Fig2

NLRP3 dependent

THP1 ATCC // THP1 NLRP3 PYD Deficient

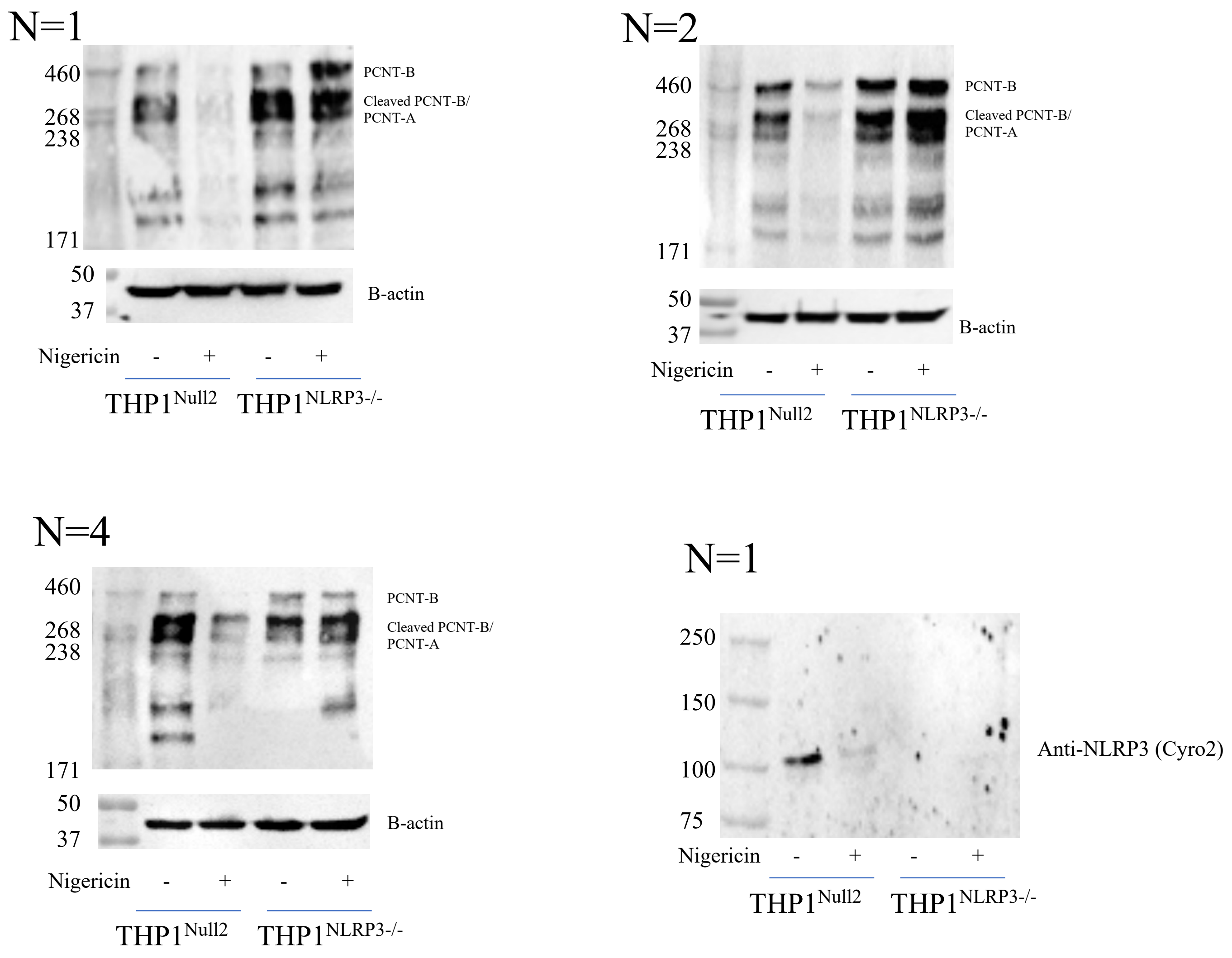

Fig2 Zvad

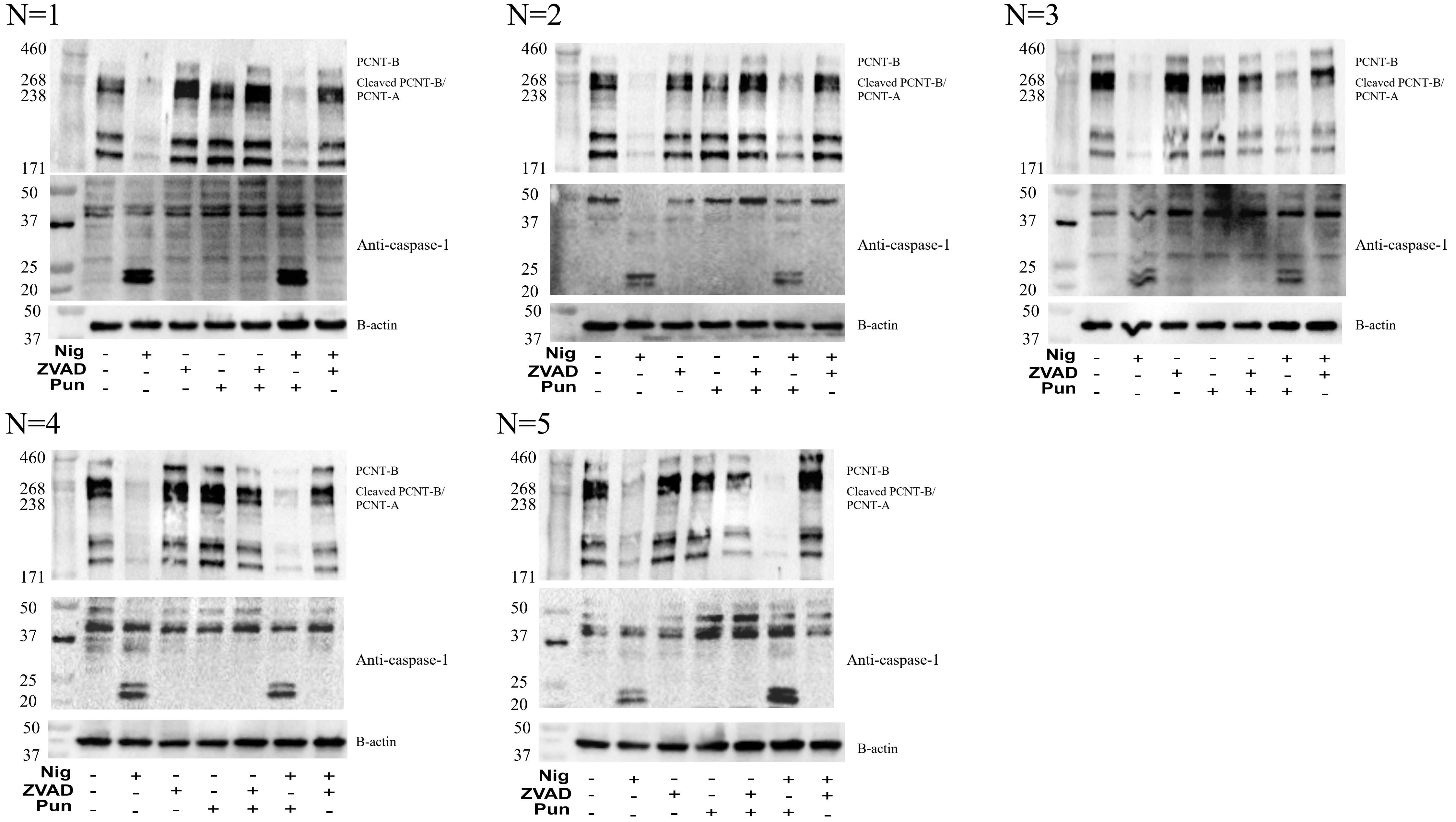
N=1
460
268
238
171
PCNT-B
Cleaved PCNT-B/
PCNT-A
50
37
25
20
Anti-caspase-1
50
37
B-actin
Nig - + - - - + +
ZVAD - - + - + - +
Pun - - - + + + -
N=2
460
268
238
171
PCNT-B
Cleaved PCNT-B/
PCNT-A
50
37
25
20
Anti-caspase-1
50
37
B-actin
Nig - + - - - + +
ZVAD - - + - + - +
Pun - - - + + + -
N=3
460
268
238
171
PCNT-B
Cleaved PCNT-B/
PCNT-A
50
37
25
20
Anti-caspase-1
50
37
B-actin
Nig - + - - - + +
ZVAD - - + - + - +
Pun - - - + + + -
N=4
460
268
238
171
PCNT-B
Cleaved PCNT-B/
PCNT-A
50
37
25
20
Anti-caspase-1
50
37
B-actin
Nig - + - - - + +
ZVAD - - + - + - +
Pun - - - + + + -
N=5
460
268
238
171
PCNT-B
Cleaved PCNT-B/
PCNT-A
50
37
25
20
Anti-caspase-1
50
37
B-actin
Nig - + - - - + +
ZVAD - - + - + - +
Pun - - - + + + -

Fig4
Yvad

N=1

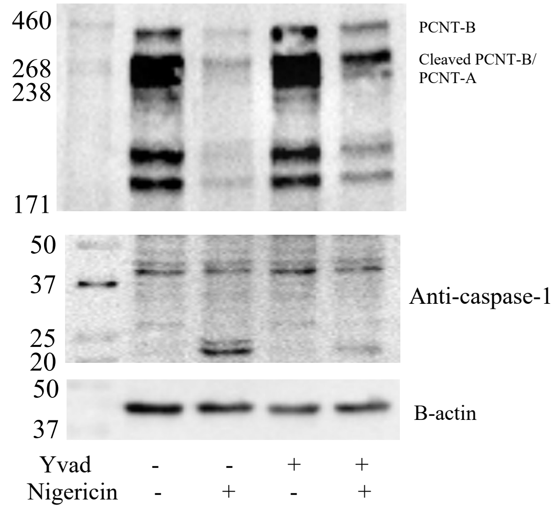

N=2

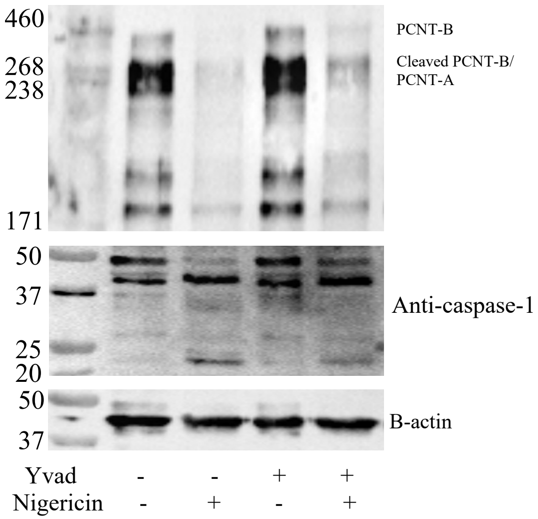

N=3

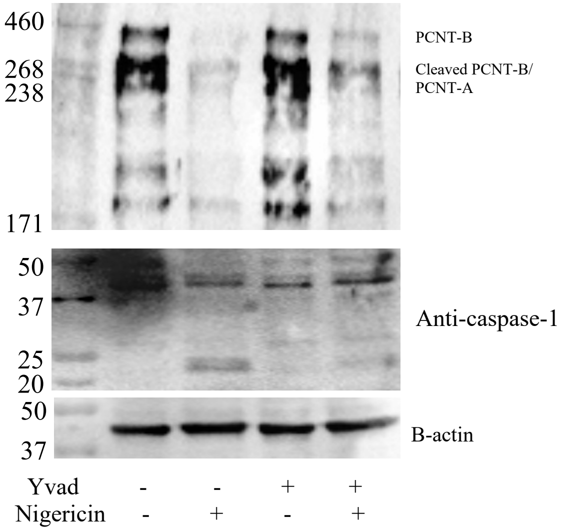

Fig3 THP1 Caspase1-/-

N=123

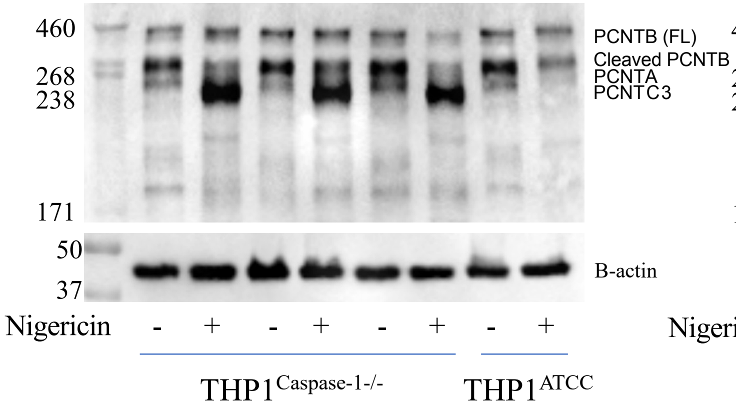

N=45

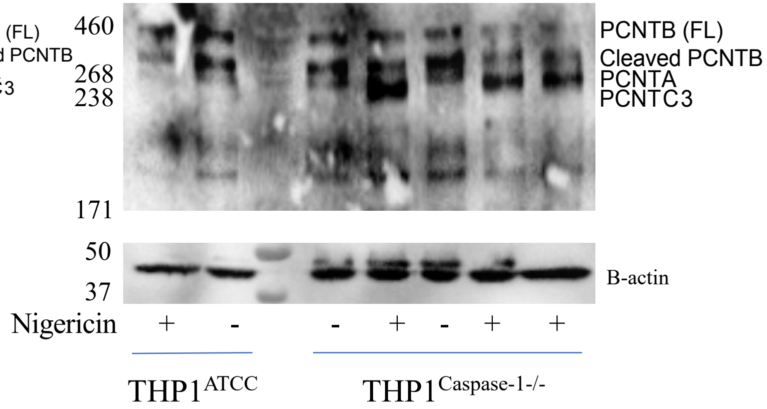

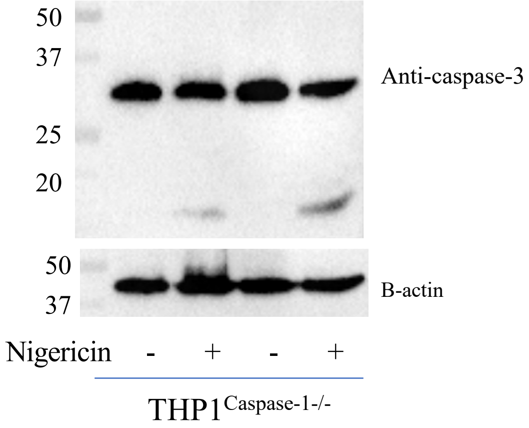

Fig4

THP1^{ATCC} and THP1$^{Caspase\text{-}1\text{-}/\text{-}}$

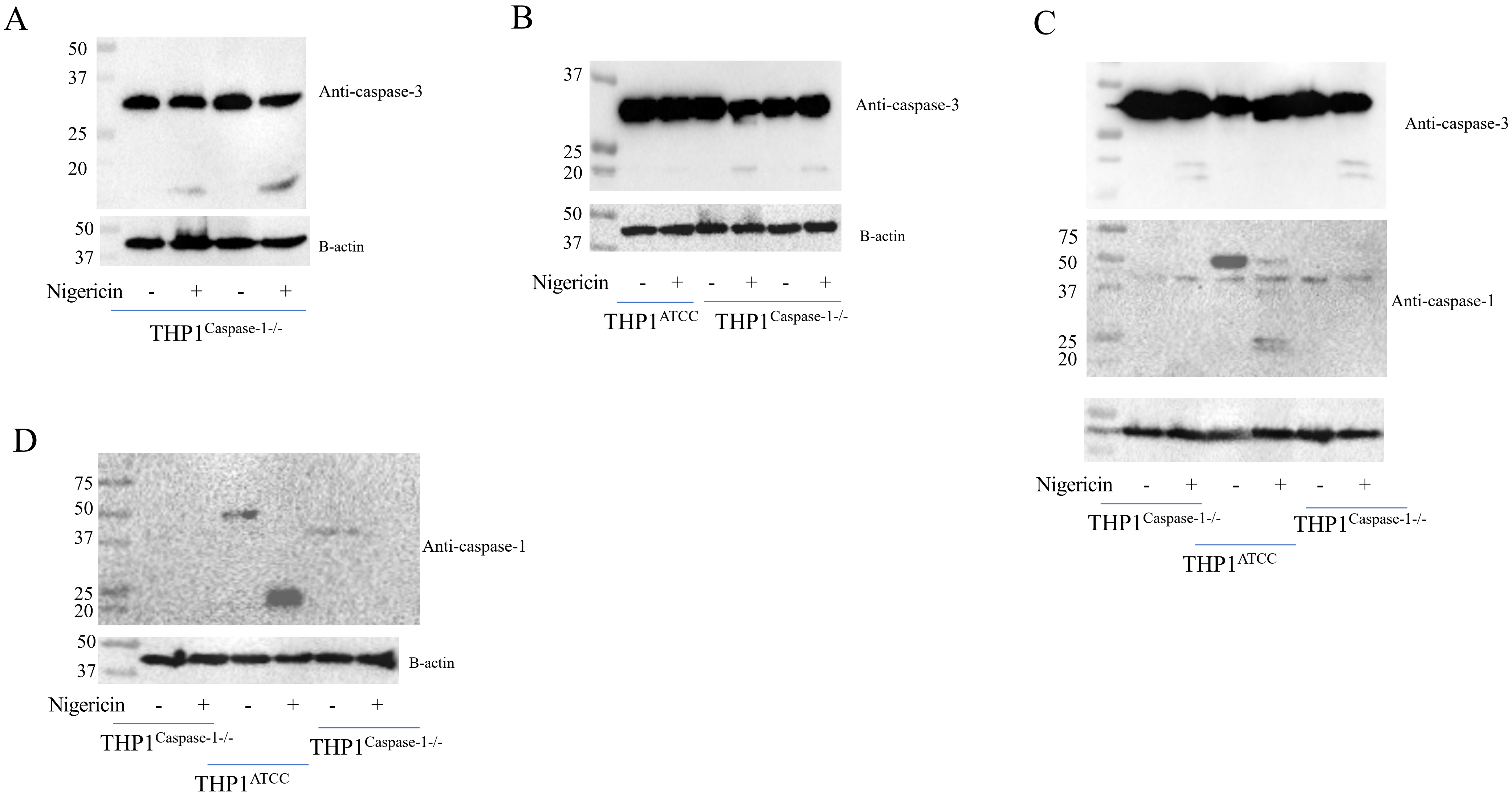

Fig5
Nig + Zvad THP1 ATCC/ THP1 GSDMD-/-

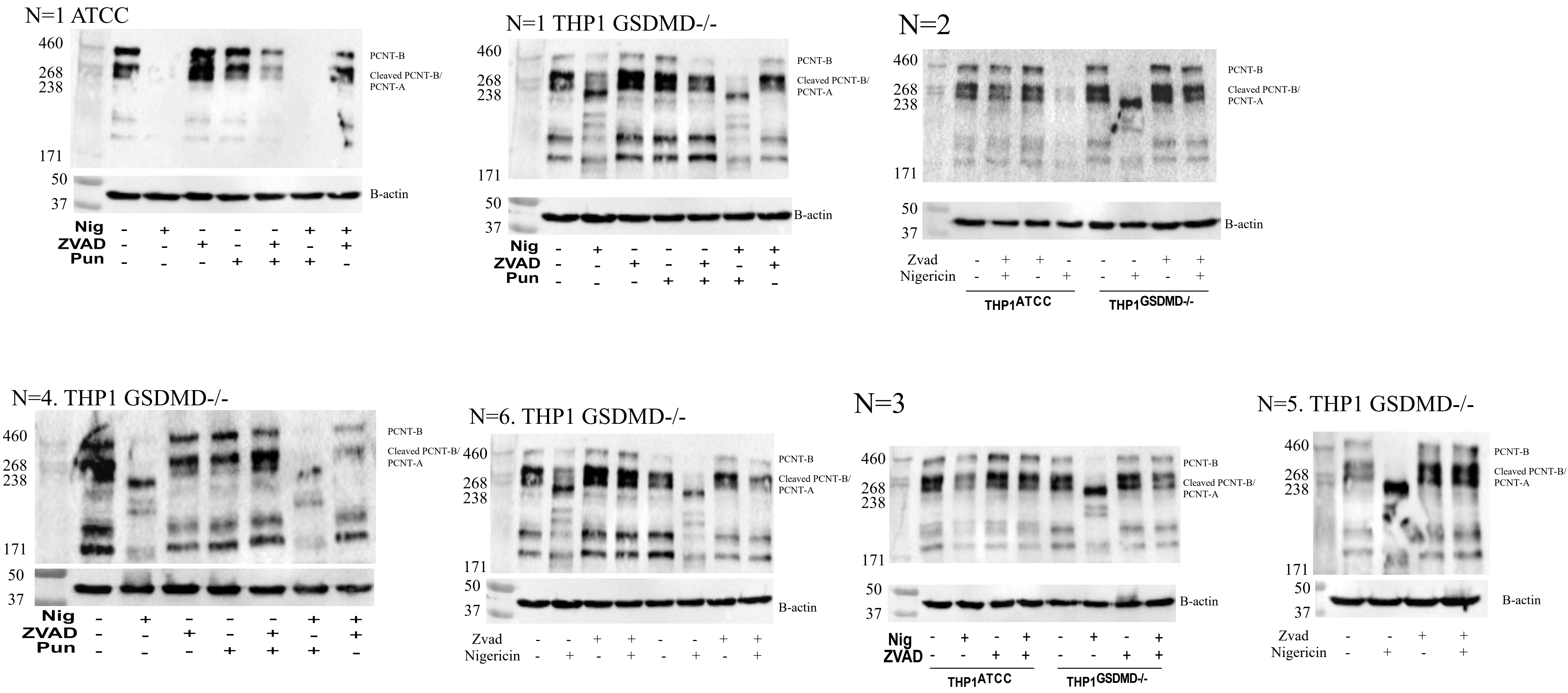

Fig5

Nig + Zvad THP1 ATCC/ THP1 GSDMD-/-

N=1

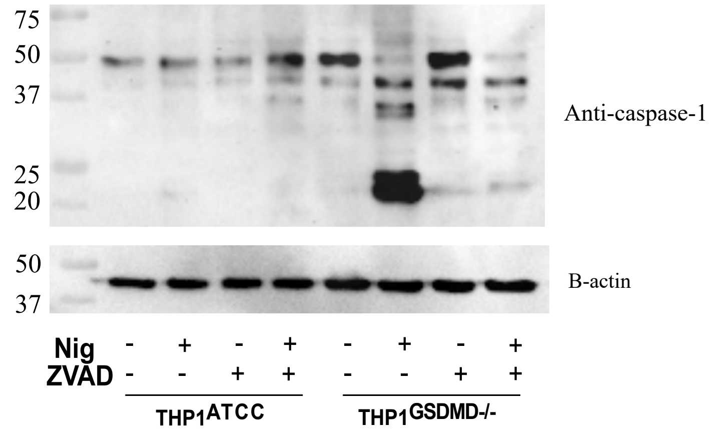

N=2

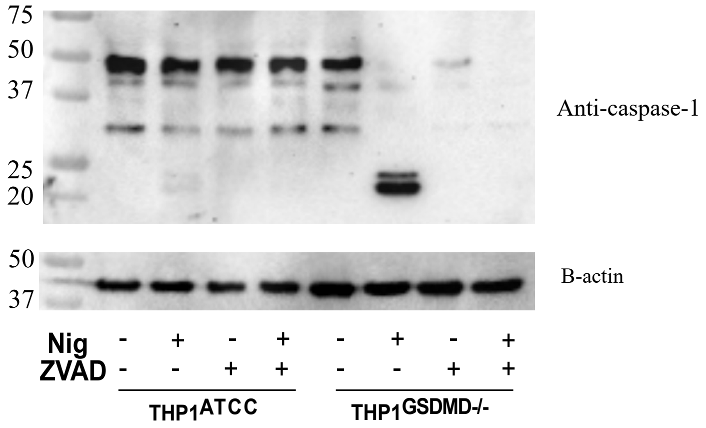

N=3

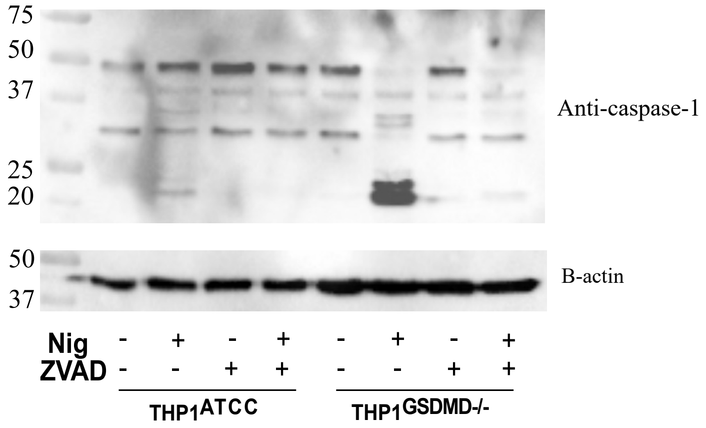

N=1

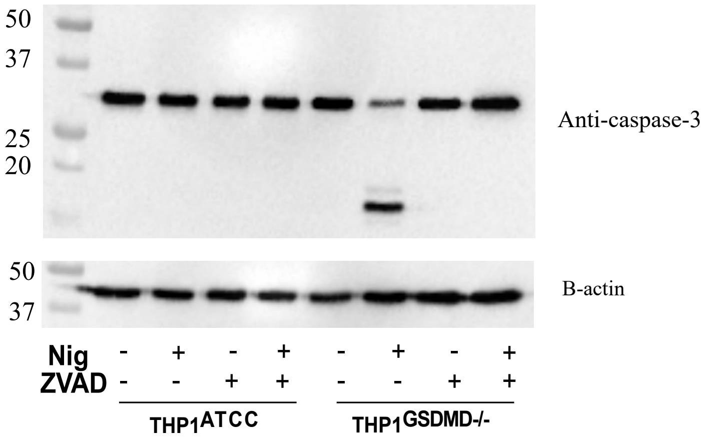

N=2

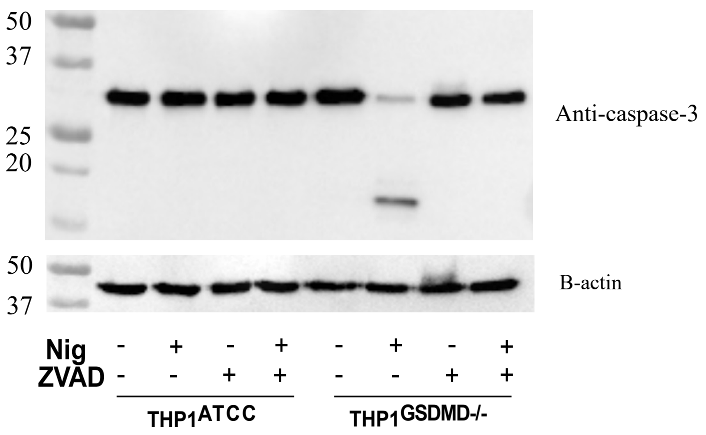

N=3

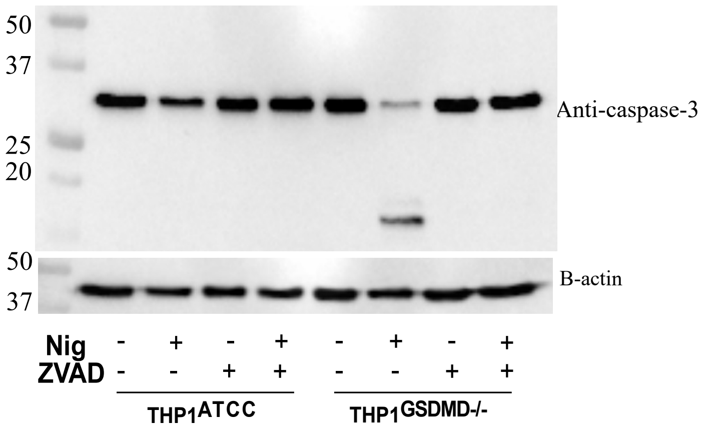

Fig5

Nig + Zvad THP1 ATCC/ THP1 GSDMD-/-

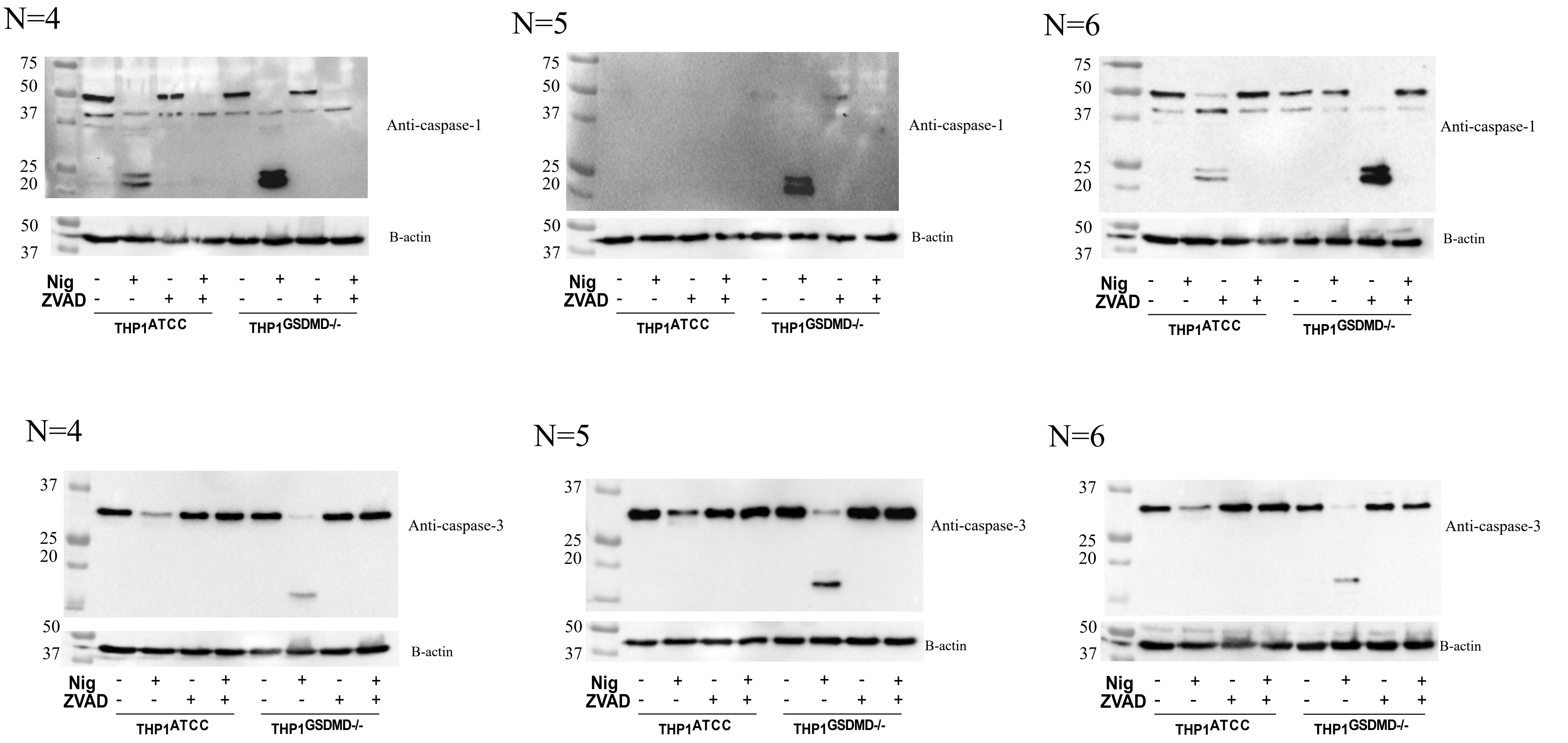

Fig5 caspase1ko cells anti cas1 and cas3
Nig + Caspase3 inhibition THP1 ATCC/ THP1 GSDMD-/-

N=1

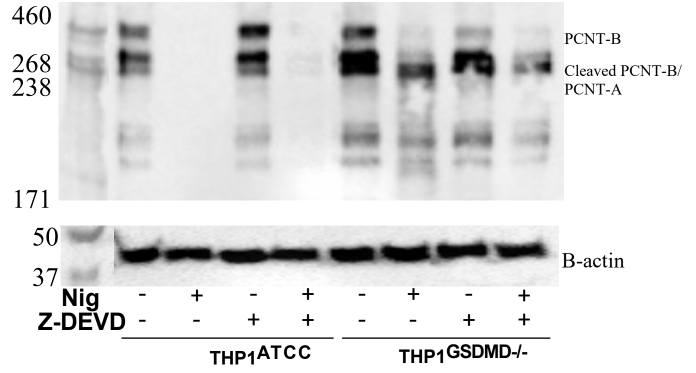

N=2

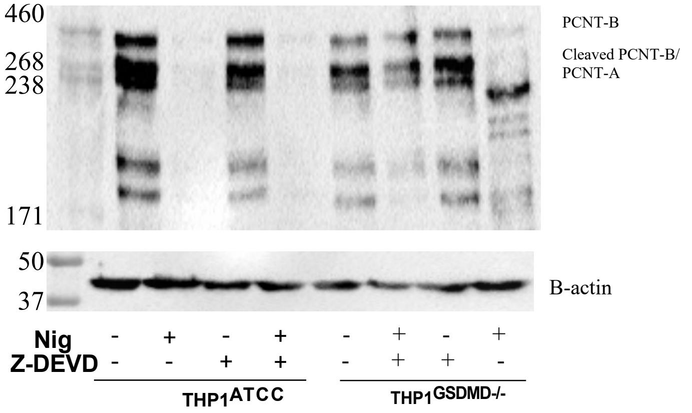

N=3

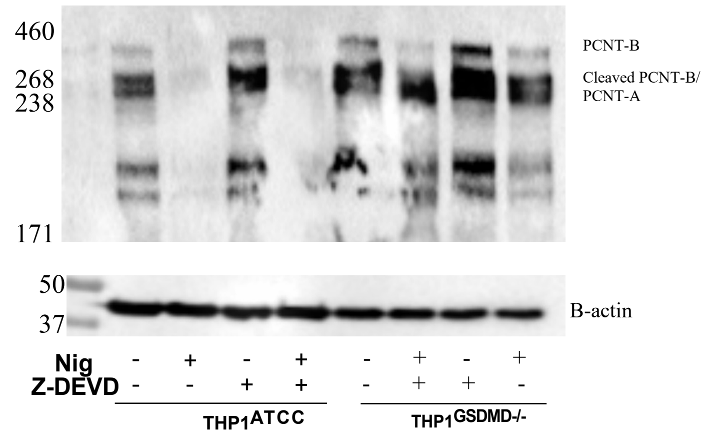

N=1

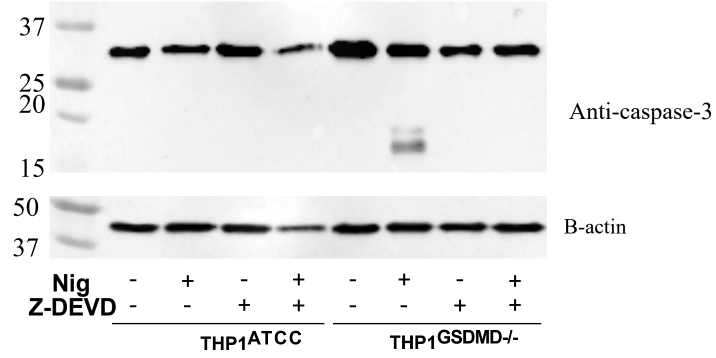

N=2

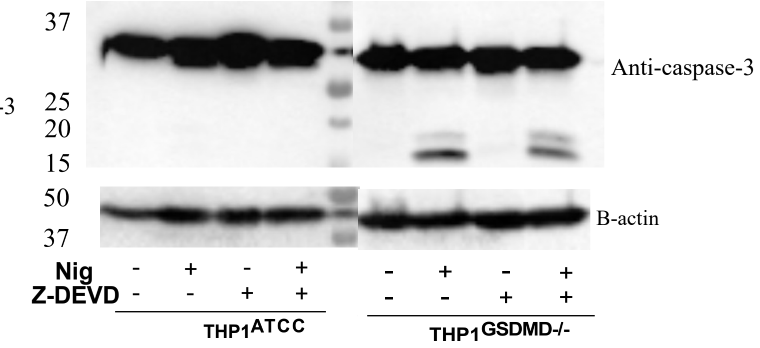

N=3

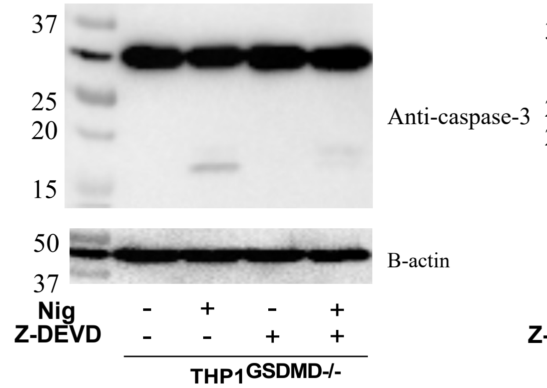

N=4

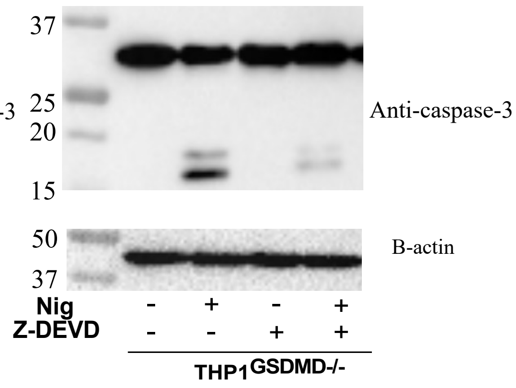

Fig6

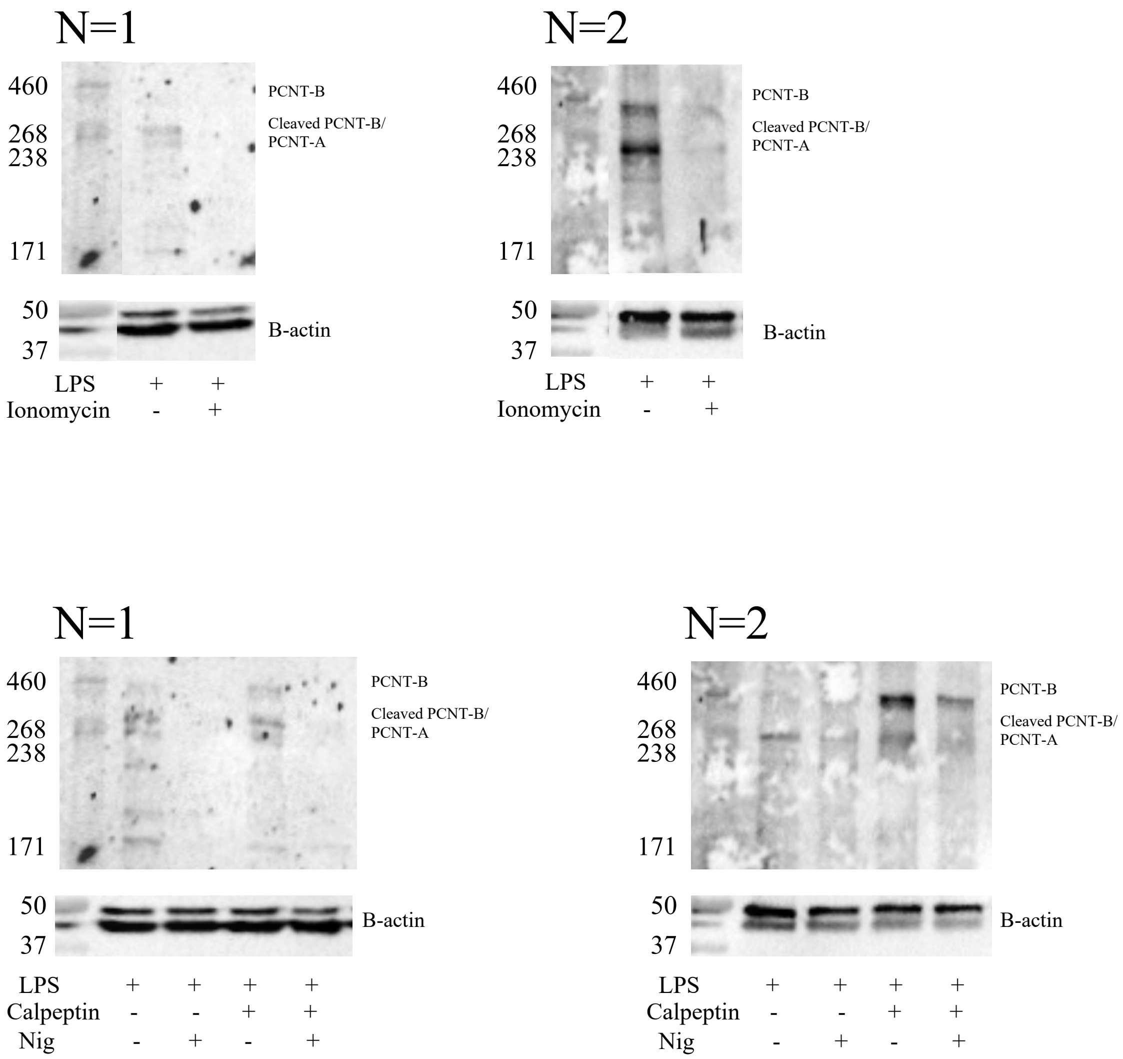
N=1
460
268
238
171
PCNT-B
Cleaved PCNT-B/
PCNT-A
50
37
B-actin
LPS + +
Ionomycin - +
N=2
460
268
238
171
PCNT-B
Cleaved PCNT-B/
PCNT-A
50
37
B-actin
LPS + +
Ionomycin - +
N=1
460
268
238
171
PCNT-B
Cleaved PCNT-B/
PCNT-A
50
37
B-actin
LPS + + + +
Calpeptin - - + +
Nig - + - +
N=2
460
268
238
171
PCNT-B
Cleaved PCNT-B/
PCNT-A
50
37
B-actin
LPS + + + +
Calpeptin - - + +
Nig - + - +

Fig7
mechanism
MG132 ATCC

N=1

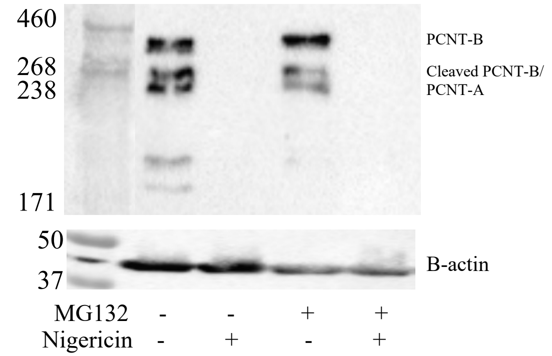

N=2

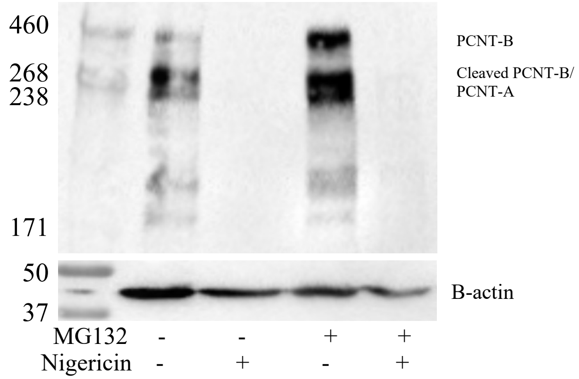

N=3

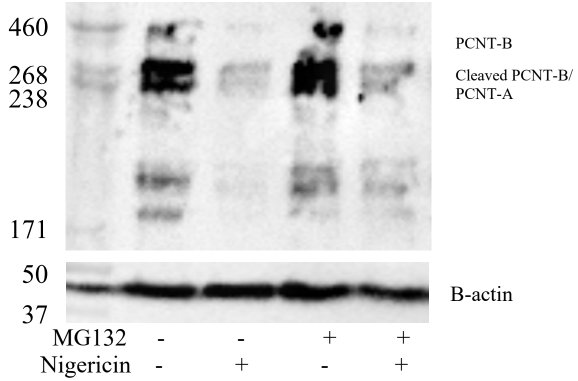

MG132 NULL2

N=1

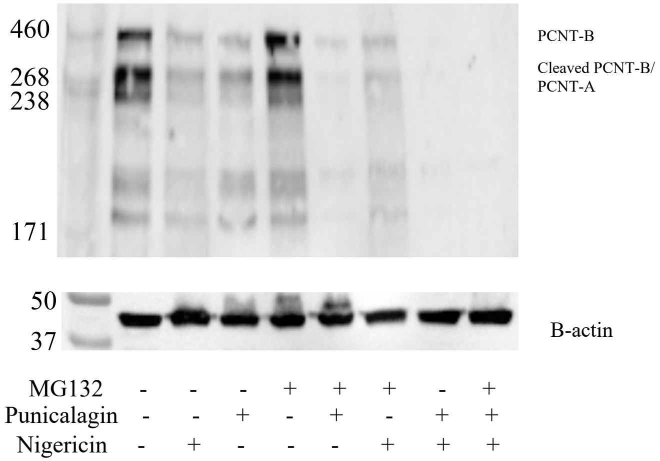

N=2

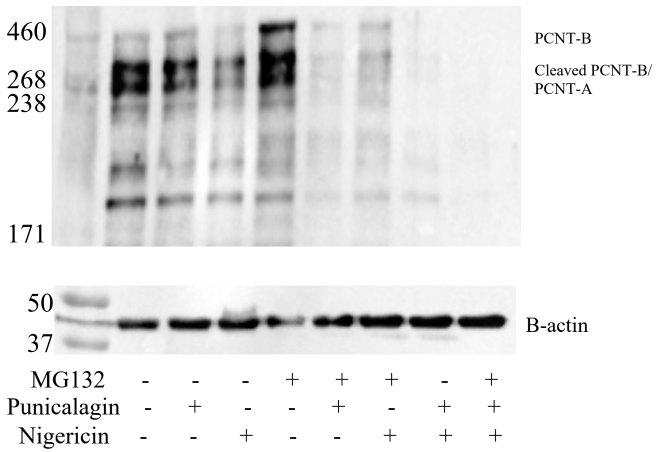

N=3

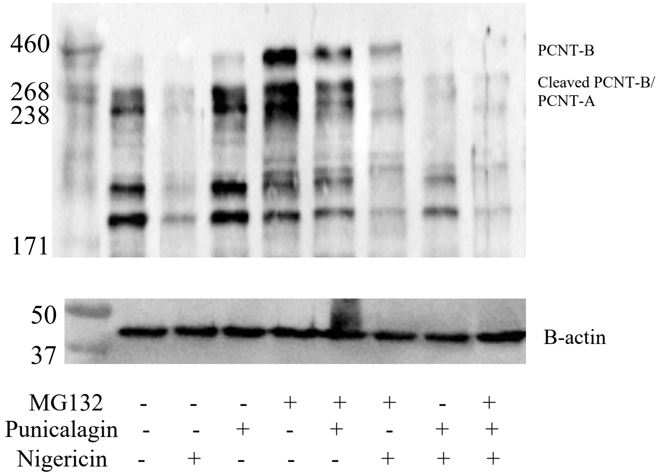

N=1 MG132 4h

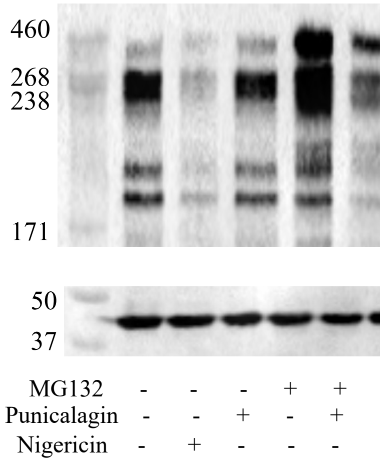

Fig7
mechanism

E-64-D

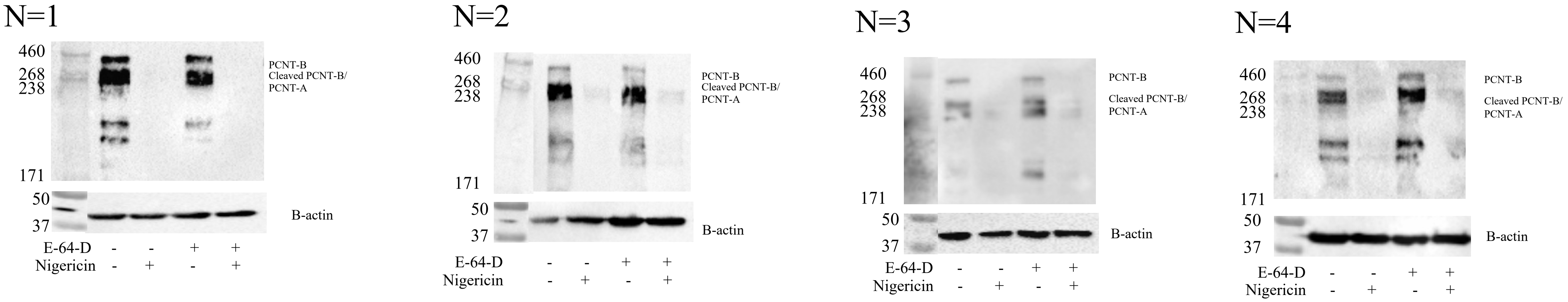

CA-074

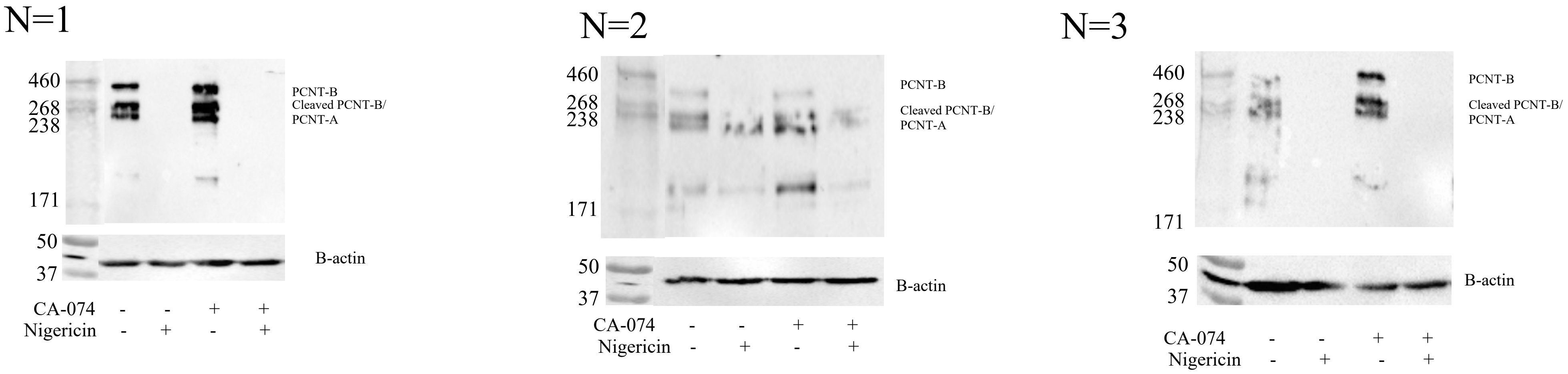

Fig7
mechanism
Pepstatin A

N=1

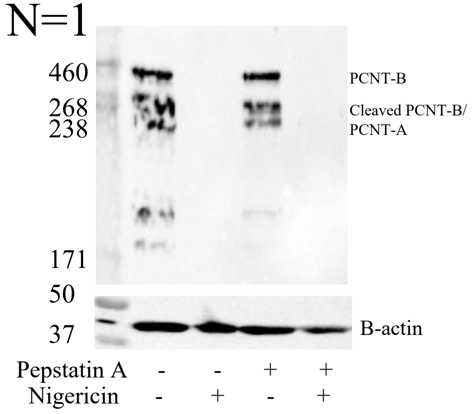

N=2

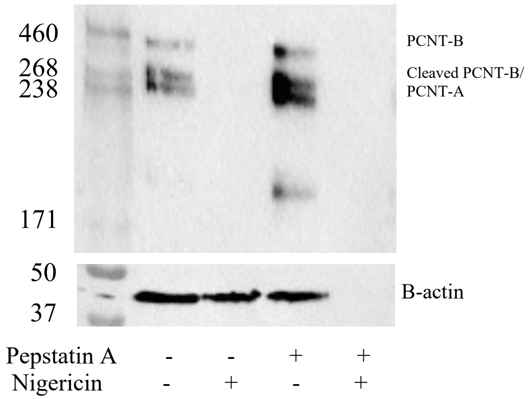

N=3

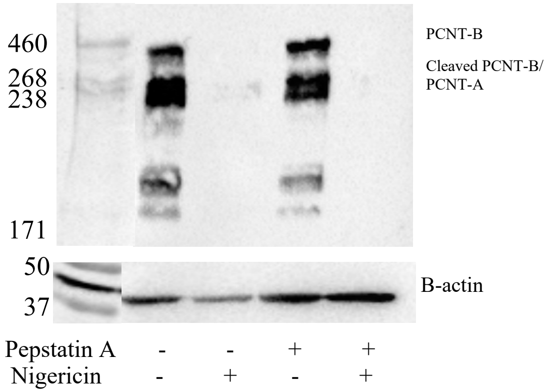

Fig7
mechanism
Bafilomycin A1

N=1

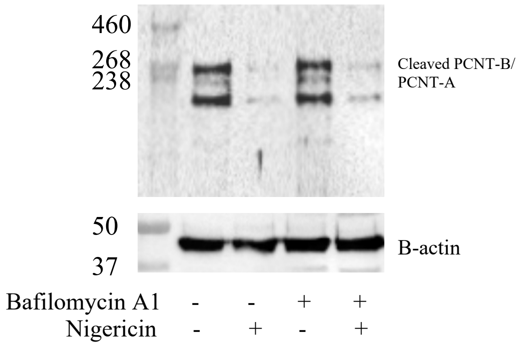

N=2

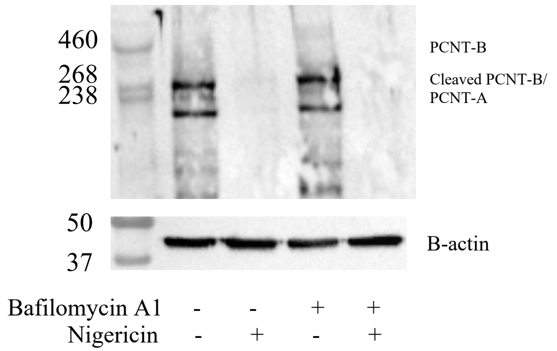

N=3

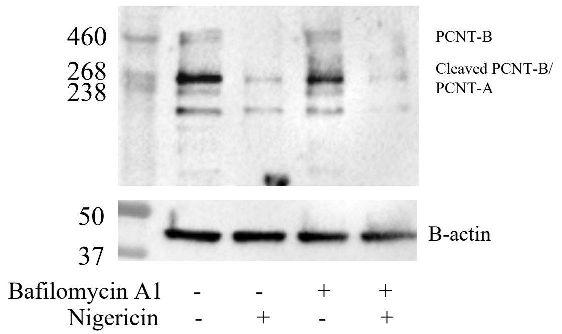

FigS1

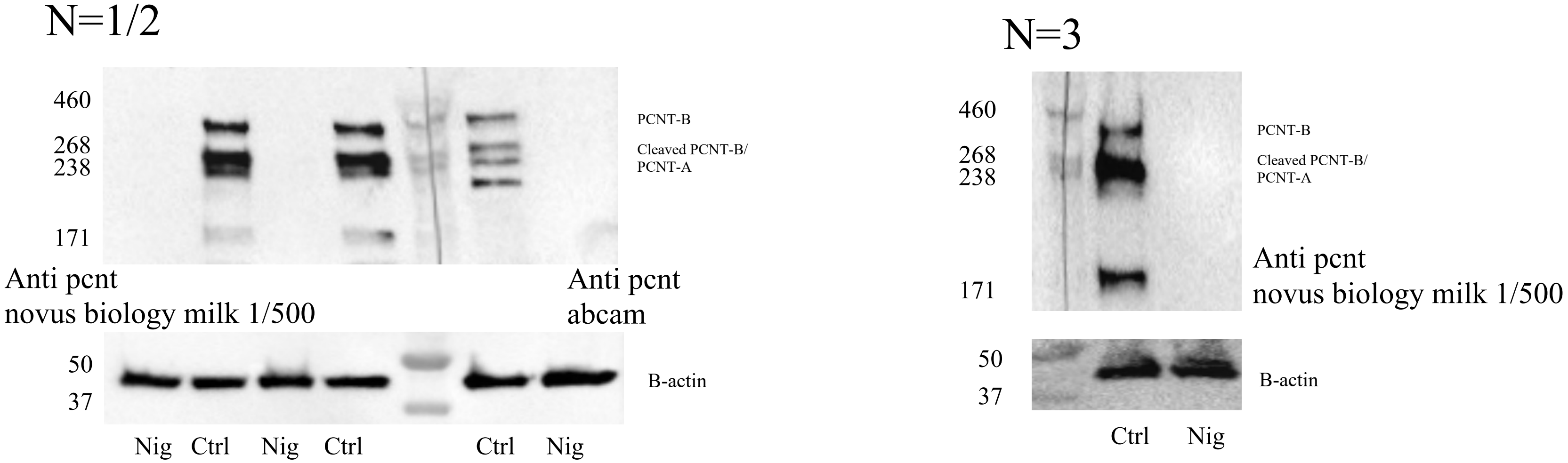
N=1/2
460
268
238
171
PCNT-B
Cleaved PCNT-B/
PCNT-A
Anti pcnt
novus biology milk 1/500
Anti pcnt
abcam
50
37
B-actin
Nig Ctrl Nig Ctrl Ctrl Nig
N=3
460
268
238
171
PCNT-B
Cleaved PCNT-B/
PCNT-A
Anti pcnt
novus biology milk 1/500
50
37
B-actin
Ctrl Nig

FigS4
LLOME

N=1

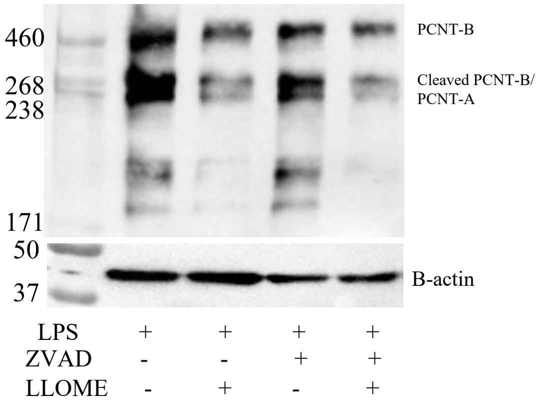

N=2

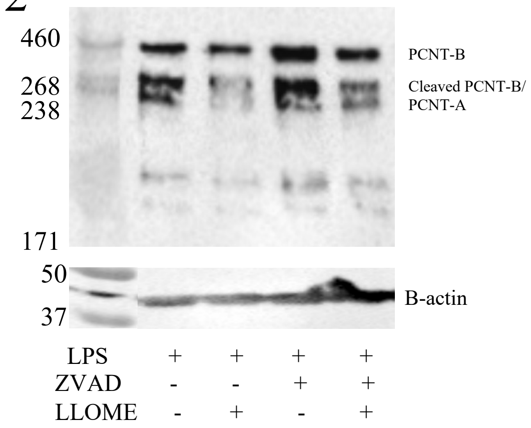

N=3

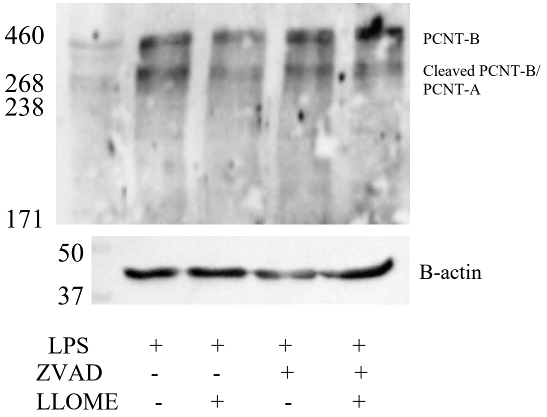

FigS4
SWELL

N=1

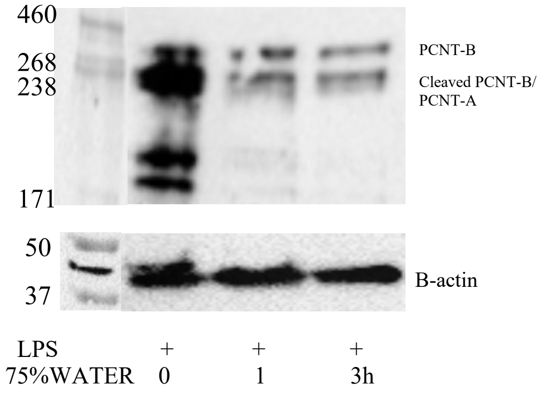

N=2

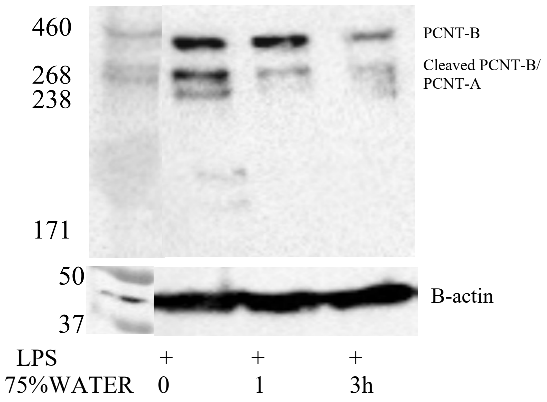

N=3

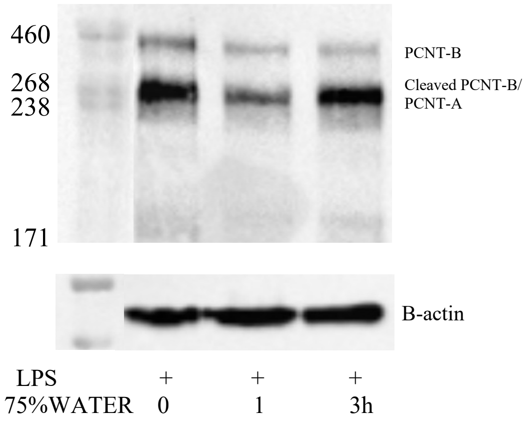

FigS4
AIM2

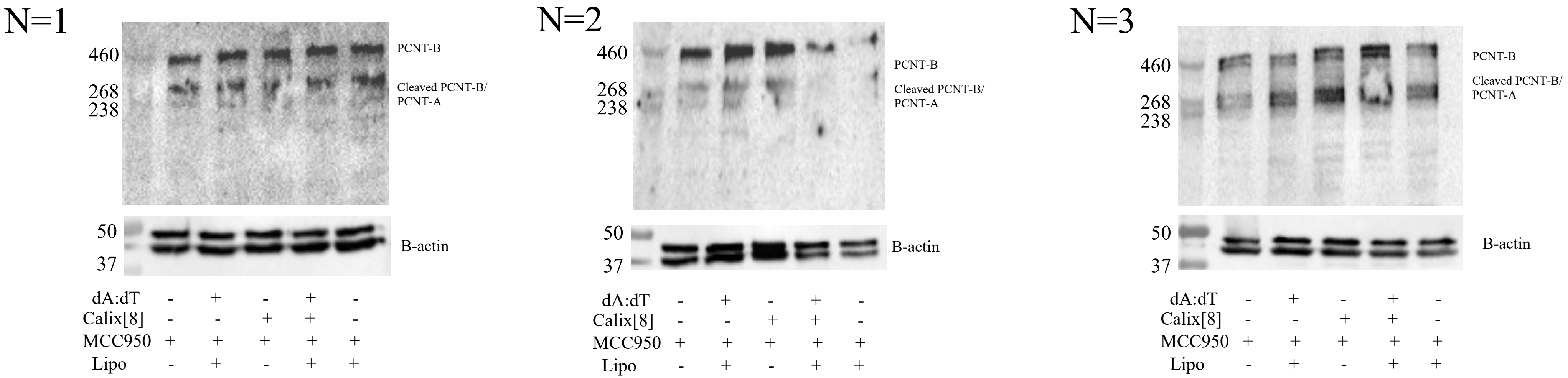

FigS4
Necroptosis

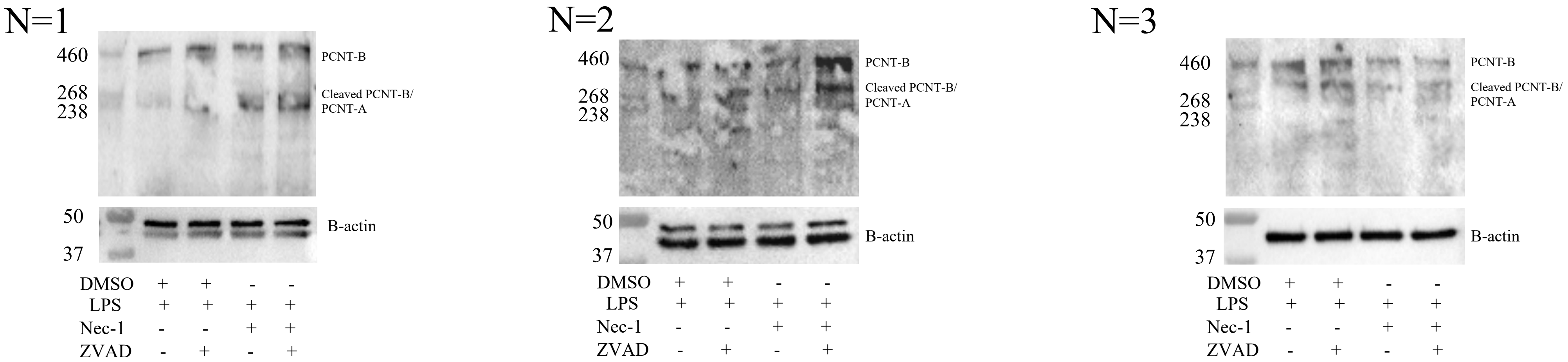

FigS5 BMDMs

N=1

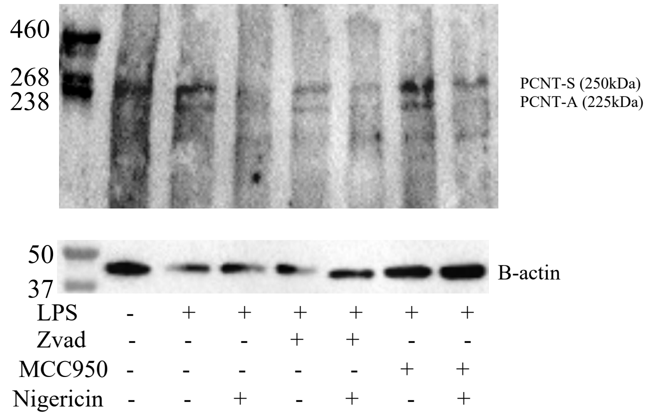

N=2

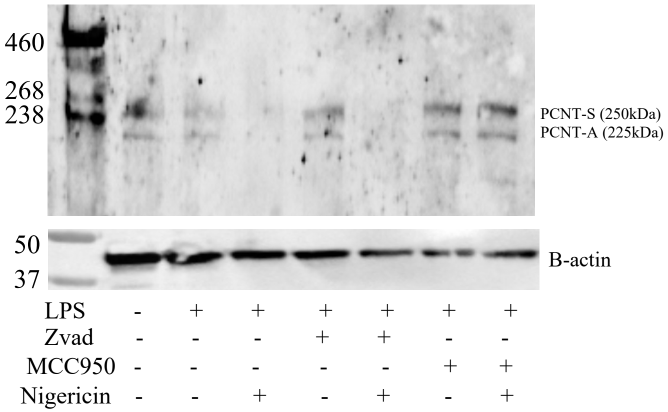

N=3

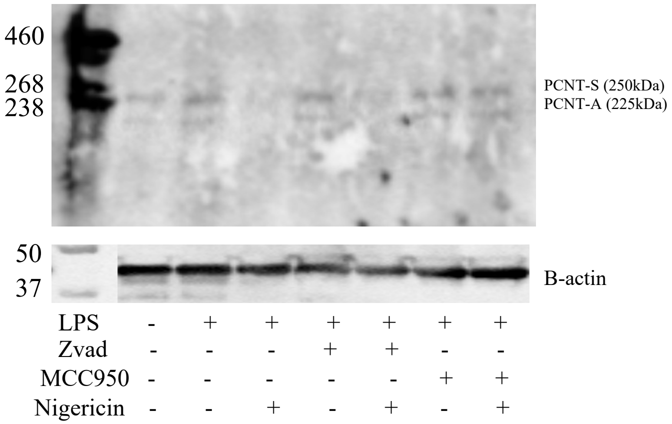

FigS7 Hek cells

N=1

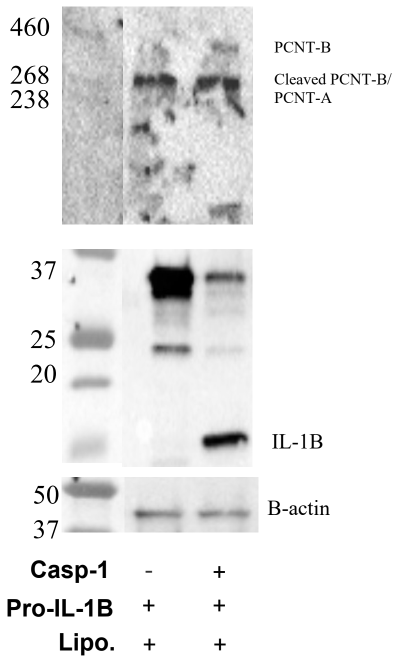

N=2

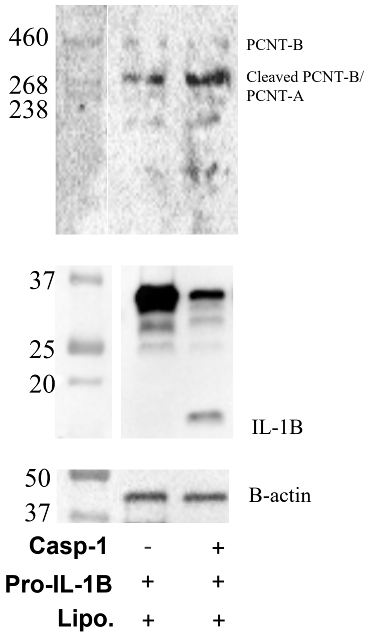

N=3

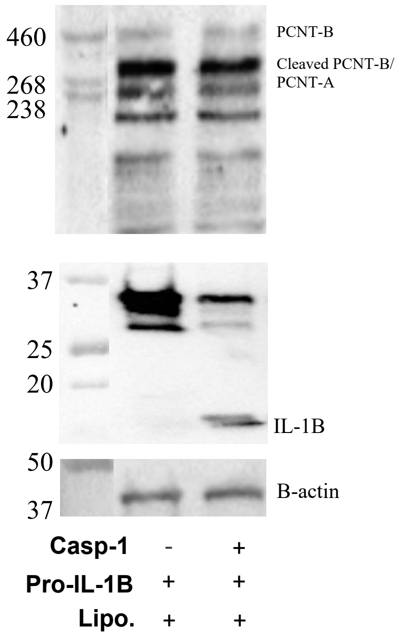

FigS8

Bafilomycin A1

N=1

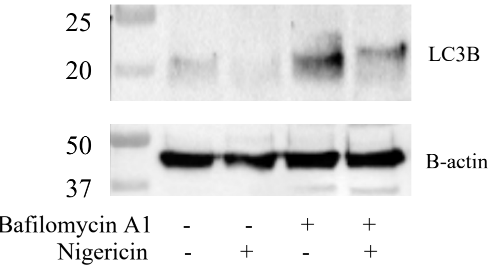

N=2

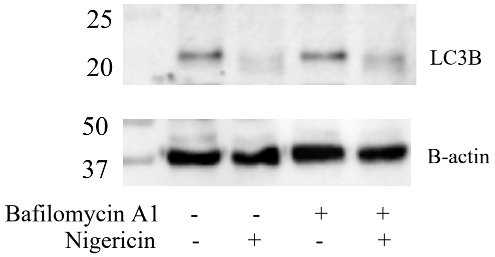

N=3

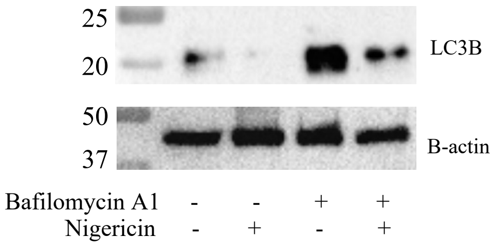

Supplement: Supplementary file 2 — Original data [file 41420_2024_2093_MOESM2_ESM.pdf]
